# A Missing Link: The Double-Slit Experiment and Quantum Entanglement

**DOI:** 10.3390/e27080781

**Published:** 2025-07-24

**Authors:** Arkady Plotnitsky

**Affiliations:** Literature, Theory, and Cultural Studies Program, Philosophy and Literature Program, Purdue University, West Lafayette, IN 47907, USA; plotnits@purdue.edu

**Keywords:** complementarity, the double-slit experiment, entanglement, experimentally quantum object, ontologically quantum object

## Abstract

This article reconsiders the double-slit experiment by establishing a new type of relationship between it and the concept of entanglement. While the role of entanglement in the double-slit experiment has been considered, this particular relationship appears to have been missed in preceding discussions of the experiment, even by Bohr, who extensively used it to support his argument concerning quantum physics. The main reason for this relationship is the different roles of the diaphragm with slits in two setups, S1 and S2, defining the double-slit experiment as a quantum experiment. In S1, in each individual run of the experiment one can in principle (even if not actually) know throughout which slit the quantum object considered has passed; in S2 this knowledge is in principle impossible, which impossibility is coextensive with the appearance of the interference pattern, once a sufficient number of individual runs of the experiment have taken place. The article offers the following argument based on two new concepts, an “experimentally quantum object” and an “ontologically quantum object.” In S1 the diaphragm can be treated as part of an observational arrangement and thus considered as a classical object, while the object passing through one or the other slit is considered as an “ontologically quantum object,” defined as an object necessary to establish a quantum phenomenon. By contrast, in S2, the diaphragm can, via the concept of Heisenberg-von-Neumann cut, be treated as an “experimentally quantum object,” defined as an object treatable by quantum theory, even while possibly being an ontologically classical object. This interaction is not an observation but a quantum entanglement between these two quantum objects, one ontologically and one experimentally quantum. This argument is grounded in a particular interpretation of quantum phenomena and quantum theory, which belongs to the class of interpretations designated here as “reality without realism” (RWR) interpretations. The article also argues that wave-particle complementarity, with which the concept of complementarity is often associated, plays little, if any, role in quantum physics, or in Bohr’s thinking, and may be misleading in considering the double-slit experiment, often explained by using this complementarity.

## 1. Introduction

This article reconsiders the double-slit experiment by establishing a new type of relationship between it and the concept of entanglement, which has been considered in connection with the experiment, but not in this way (e.g., [1,2] and further references there). This link, which is the missing link of my title, appears to have been missed in preceding discussions of the experiment, including in earlier treatments of the experiment by this author, most recently [3], from a perspective otherwise similar to that of this article. It also appears to have been missed by N. Bohr, who extensively used the double-slit experiment to support his argumentation concerning quantum physics. Bohr might have been aware of the main reason for this relationship, which is the different status of the diaphragm with slits in two setups, S1 and S2, defining the double-slit experiment as a *quantum* experiment. In S1, referred to as the “which-way” setup, in each individual run of the experiment one can in principle (even if not actually) know through which slit the quantum object considered has passed; in S2 this knowledge is in principle impossible, which impossibility is coextensive with the appearance of the interference pattern, once a sufficient number of individual runs of the experiment have taken place.

The article’s argument is based on two new concepts—an “ontologically quantum object” and “experimentally quantum object” and three postulates, defined below. An ontologically quantum object is an object necessary for the occurrence of a quantum phenomenon, observed in a measuring instrument as an effect of the interaction between this object and this instrument. Representative examples of ontological quantum objects are elementary particles, such as electrons, photons, or neutrinos, although ontological quantum objects can be composite and even macroscopic, such as Josephson devices. An experimentally quantum object is an object that can, as an object of investigation, be treated by means of quantum theory, even while being an ontologically classical object, an object treated by means of classical physics, when considered by itself. Prominent examples of experimentally quantum objects in foundational discussions of quantum physics are “the cat” in Schrödinger’s cat experiment or “the friend” in Wigner’s friend experiment, or, as I shall argue here the diaphragm with slit in S2 of the double-slit experiment, in the interpretation of these experiments adopted here and discussed in connection with the first two experiments in [4,5]. The treatment of such objects by means of quantum physics is possible by virtue of the Heisenberg-von-Neumann cut, explained later in this article. An ontologically classical object can, however, only be considered as an experimentally quantum object if it interacts with an ontologically quantum object, such as that traversing the diaphragm with slits. An ontologically quantum object can be an experimental quantum object if considered by itself, but an ontologically classical object cannot. In the present view, although ultimately composed of ontologically quantum objects, such as elementary particles, an ontologically classical object cannot be assumed to be an ontologically quantum object. (There are arguments to the effect that all objects considered in quantum physics can be assumed to be ontologically quantum.) Nor, conversely, can an ontologically quantum object be an ontologically classical object. While an ontologically quantum object can, in certain circumstances, be treated by classical physics, it retains its ontologically quantum nature and, as such, can disrupt this classical treatment. Ontologically classical objects will hereafter be referred to as classical objects, unless they are considered as experimentally quantum. The article is also a contribution to the question of the nature of quantum objects, widely discussed in foundational literature on quantum theory. One of the novel aspects of this article in this connection is considering the entanglement between ontologically quantum objects and classical objects, treated as experimentally quantum objects, otherwise than in measurements, which have been considered as this type of entanglement beginning with E. Schrödinger’s introduction of the concept [6] (pp. 161–163).

The article offers the following argument: In S1 the diaphragm can be treated as part of an observational arrangement and, as such, along with the rest of this arrangement (such as counters registering through which slit the quantum object considered passed), is assumed to be an ontologically classical object, treated by classical physics. By contrast, in S2, the diaphragm can, by the Heisenberg-von-Neumann cut, be treated as an experimentally quantum object interacting in each run of the experiment, with an ontologically quantum object, O, such as an electron, traversing it. This interaction is not an observation, as in S1, but is a quantum entanglement between these two quantum objects, one, the diaphragm, experimentally quantum, and the other, the object passing the diaphragm, ontologically quantum. As in any entanglement, neither system can be defined separately at the time of their interaction, but only before or (by disentangling the entanglement) after this interaction, for example, when the quantum object that had traversed the diaphragm hits the screen. At this stage, the diaphragm is treated as a classical object, with its observable state remaining that of the diaphragm with two slits in their specific positions.

This argument is grounded in a particular interpretation of quantum phenomena and quantum mechanics (QM), or quantum field theory (QFT), that responds to this situation. These are the only two quantum theories to be considered here and the term quantum theory (QT) will, unless qualified, only refer to them. (Alternative quantum theories, such as Bohmian mechanics, will only be mentioned in passing.) The present interpretation belongs to the class of interpretations designated as “reality without realism” (RWR) interpretations, to which Section 2 is devoted. These interpretations were considered by this author previously, most extensively in [7]. They place the ultimate reality responsible for quantum phenomena beyond representation by QM, or QFT, or otherwise, or even beyond conception. This assumption will be designated here as the Heisenberg postulate, because, while it has precursors, specifically Bohr’s concept of “quantum jump” in his 1913 atomic theory, it was made by Heisenberg in his invention of QM.

I also argue that wave-particle complementarity, with which the concept of complementarity has been commonly associated, is unnecessary, if applicable at all, in quantum physics and can be misleading in considering the double-slit experiment, often explained in terms of this complementarity. The idea of wave-particle duality, which should not be confused with wave-particle complementarity, is even more questionable and, in RWR interpretations, inapplicable in quantum physics and hence in the double-slit experiment, sometimes still considered in terms of this duality as well. In my view, wave-particle complementarity did not play a significant, if any, role in Bohr’s interpretation either, especially in its ultimate RWR version, as against other forms of complementarity, in particular that, correlative to the uncertainty relations, of the simultaneous (exact) position and momentum measurements. Bohr, while using de Broglie’s *formula* for matter waves, rejected the idea of wave-particle duality, following the introduction of QM. It is important to keep in mind that Bohr changed his views, sometimes significantly, several times, as discussed by this author in detail in [8]. Bohr’s ultimate, RWR, interpretation was introduced in the late 1930s, following a decade of the development of his views, which requires one to specify to which version of his interpretation one refers. Here, I shall primarily focus on his ultimate interpretation, unavoidably in the present interpretation of it, and unless qualified, “Bohr’s interpretation” will refer to this interpretation. There are other interpretations of Bohr and his evolving views, some of which offer different assessments of the role of wave-particle complementarity (or duality) in Bohr’s thinking, for example, in several instructive articles assembled in [9], which contains further references concerning this subject. Such alternative views, however, do not impact my argument against wave-particle complementarity (or duality), which would apply even if one saw Bohr’s thinking, at some or all stages, as assuming wave-particle complementarity (or duality). The designation “the Copenhagen interpretation” requires even more qualifications, beginning with whose interpretation it is. I shall avoid this designation altogether.

The double-slit experiment does not depend on QM, the main form of QT to be considered here. QM, however, correctly predicts the data observed in the experiment, keeping in mind that photons are relativistic objects and require quantum electrodynamics (QED) to be properly treated. Rather than only with light, with which it was concerned (as a classical experiment, before A. Einstein introduced the concept of photon in 1906, and then as a quantum one), the double-slit experiment can in principle be performed with any type of (ontologically) quantum objects, although there are practical limits for doing so. The experiment was first performed as a quantum experiment with anything other than light, specifically electrons, only in the 1960s. It had functioned previously as a thought-experiment, without much doubt, that it could in principle be performed on any type of quantum objects, following L. de Broglie’s conjecture in 1923 that wave-particle duality would apply to all elementary constituents of nature. The conjecture was quickly confirmed by the discovery of electrons’ diffraction in crystals. The diffraction is not the same as interference, which, rather than merely a diffraction by a slit, defines the double-slit experiment as a quantum experiment. As a classical experiment, Thomas Young’s double-slit experiment with light has been around since 1801, when it was performed to resolve the dispute whether light was composed of particles, according to Newton’s corpuscular theory, or was formed by waves traveling through some form of ether. The interference patterns found in the experiment, *as a classical experiment*, appeared to have answered the question in favor of the wave theory. The latter had remained dominant until M. Planck’s 1900 discovery of the black body radiation law, which ushered in quantum physics, brought this question back to the center stage of fundamental physics.

The double-slit experiment does have its rivals in illustrating the strange features, “mysteries,” of quantum physics. Among them are the Stern-Gerlach experiment, various experiments in quantum interferometry (e.g., using the Mach–Zender interferometer), experiments with half-silvered mirrors, and the EPR (Einstein-Podolsky-Rosen) type experiments. The latter, especially in D. Bohm’s version (dealing with discrete variables), have been widely used in foundational discussions of quantum physics during the last half a century, following Bell’s theorem and related developments. The double-slit experiment, however, retains its reputation as, in N. D. Mermin’s phrase, “the greatest of all quantum conundrums” [10] (p. 108).

There are several advantages of the double-slit experiment that help this fame. It can be easily explained qualitatively without any technical knowledge of quantum physics. This made it ubiquitous in popular literature. It nicely illustrates the uncertainty relations and the complementarity of *exact* position and the momentum measurements, in the two corresponding setups of the experiment, S1 and S2. Such measurements are always mutually exclusive and hence cannot be performed jointly, which is a defining aspect of Bohr’s concept of complementarity. On the other hand, which is an equally defining part of Bohr’s concept, one has a choice to perform either one or the other at any moment of time. The *either-or* of complementarity and the freedom of choice in establishing either one or the other complementary phenomenon defines the fundamental difference between the complementarity and the duality of the features considered, say, X and Y, such as the wave-particle duality. This duality is always defined by *both* X and Y, rather than by *either* X *or* Y. Considering one or the other component of a duality merely amounts to selecting one or the other aspect of an already existing reality, rather than, as in the case complementarity, to establishing one or the other reality by interacting with nature by means of experimental technology. The duality view is far from having been abandoned (even apart from Bohmian mechanics, where it is inherent), and it figures in the treatment of the double-slit experiment, including in connection with entanglement [1,2]. The use of wave-particle duality in quantum physics is, I argue, questionable and is precluded in RWR interpretations, which, I also argue, do not require wave-particle complementarity either. The double-slit experiment is also well suited for exhibiting the irreducibly probabilistic or statistical nature of quantum predictions, and the coexistence of random individual events and the correlational order of their collective organization in certain specific circumstances, a combination that is one of the greatest mysteries of quantum physics. These correlations are more commonly discussed in the EPR-type experiments, but they are also found in the “interference” pattern, which is, in effect, a correlational pattern observed in the double-slit experiment. R. Feynman thought that the double-slit experiment “contains the only mystery” of quantum physics, defying our capacity to understand how quantum phenomena are possible [11] (v. 3, 1.1–1.8).

Properly predicting the quantitative data observed in actually performed double-slit experiments does, of course, require a theory, and QM (or QED in the case of photons) does so with great accuracy. This fact helps to mitigate the unfortunate connotations of the word “mystery,” often used in considering the double-slit experiment or quantum physics. As emphasized by Bohr, QM, or QFT, is a “rational” theory free from any “mysticism incompatible with the true spirit of science” [12] (p. 83) [13] (v. 2, p. 63). One might say that quantum physics has mysteriousness without mysticism [7] (pp. 96–97). Mysticism would assume some divine or divine-like agency responsible for the reality considered, even if this agency cannot be assigned any conceivable attributes, as in the so-called mystical or negative theory. One need not, however, make this assumption, even if one cannot conceive of how quantum phenomena are possible or why QT predicts them fully in accord with quantum experiments, as things stand now, a crucial qualification, assumed throughout this article. This is what gives quantum physics its mysteriousness: this mysteriousness is not in how QT works but in the fact that it does work.

Feynman also said, equally famously, that “nobody understands quantum mechanics” [14] (p. 129), a statement endlessly cited and often misunderstood. It is not that it does not reflect Feynmann’s concerns with QM or QFT, to which he made many major contributions, earning him a Nobel prize (for the renormalization of QED), shared with S-I. Tomonaga and J. Schwinger. Feynman did have such concerns. He made his statement in juxtaposing it to, and, rightly, denying, the adage that only a few people (he mentioned twelve) understood Einstein’s relativity. I would argue, however, that Feynman’s meaning was not that one cannot understand the mathematical or experimental nature of QM (or QFT), which he himself understood very well. He meant that nobody could explain or even imagine how quantum phenomena come about; thus, in accord with RWR interpretations, which assume this impossibility as a postulate, the Heisenberg postulate.

I am not saying that Feynman subscribed to an RWR interpretation. He appears to have been uneasy about this type of interpretation, even if he did not deny that it is possible. Einstein, the main figure and a symbol of the discontent with QT, did not deny this possibility either and even appears to have seen RWR interpretations, such as that of Bohr, virtually inherent in QM or QFT. Einstein said that this kind of interpretation, which also entails an irreducibly probabilistic or statistical nature of QM, if assumed to be a complete theory, is “logically possible without contradiction” but “is so very contrary to [his] scientific instinct that [he could not] forego the search for a more complete conception” [15] (p. 375). By the latter Einstein meant a theory of quantum phenomena obeying his realist imperative for a fundamental physical theory. This imperative means that a complete theory offers a mathematized representation of how the phenomena considered come about, rather than only predicting them, and moreover, only probabilistically. Bohr, citing Einstein’s statement, replied in his 1949 article as follows:

Even if such an attitude might seem well balanced in itself, it nevertheless implies a rejection of the whole argumentation exposed in the preceding [presenting Bohr’s interpretation], aiming to show that in quantum mechanics, we are not dealing with an arbitrary renunciation of a more detailed analysis of atomic phenomena but with a recognition that such an analysis is *in principle* excluded.

[13] (v. 2, p. 62)

In other words, Einstein’s attitude implied a rejection, on philosophical grounds, of an RWR-type interpretation (assumed by Bohr here), even if it was “logically possible without contradiction,” thus without offering a counterargument to it. While it is possible to see Bohr as making a stronger than interpretive claim, I would read this statement as only asserting that an RWR interpretation is “logically possible without contradiction,” while allowing for other interpretations. In any event, this is the view adopted in this article.

## 2. Quantum Theory and Reality Without Realism

### 2.1. The Heisenberg, Bohr, and Dirac Postulates

Physics, as a science, consists of both establishing physical phenomena, in which case one refers to experimental physics, and investigating these phenomena by theoretical means, in which case one refers to theoretical physics. Both relativity theory, special and general, and QM and QFT are parts of theoretical physics in their respective domains. While, sometimes, QT that deals only with discrete variables, such as spin, and finite-dimensional Hilbert spaces, over C, is distinguished from QM, in this article it will assumed to be part of QM, as a theory dealing with both discrete and continuous variables, such as coordinates, momenta, or energy, which require infinite-dimensional Hilbert spaces, over C. “Classical physics” also commonly refers to classical physical theories, such as classical mechanics, classical statistical physics, chaos theory, or classical electromagnetic theory. I shall retain this use, qualifying when referring to experimental classical physics.

Technically, a different interpretation of a theory forms a different theory, because an interpretation may involve concepts not shared by other interpretations. For simplicity, however, I shall refer to different interpretations of QM or QFT, or QLTs. While quantum phenomena can be interpreted independently of any theory, commonly, including in this article, an interpretation of a QT, such as QM or QFT, encompasses both this theory and quantum phenomena. By contrast, in the case of classical physics and relativity, I shall, as is customary, refer to theories themselves. (Relativity is not only a theory either, and contains relativistic phenomena that could in principle be explained by another theory.) This is because most interpretations of these theories, including the ones assumed here, are realist, insofar as the reality responsible for the phenomena considered is assumed to be represented by these theories. Sometimes realist theories or interpretations, including those of quantum physics, are also referred to as ontological or ontic, from the ancient Greek “*ontos*,” designating being. In this article, “ontological,” as in “ontologically quantum objects,” will refer to something that is real or exists, without necessarily being representable or even conceivable, and ontologically quantum objects are not in RWR interpretations. There are departures from realism in interpreting classical physics or relativity, but they are rare, and never quite of RWR type. By contrast, the proliferation of different and sometimes incompatible interpretations of QM or QFT has been massive, and debates concerning them have continued for a century now. There are also alternative interpretations of the double-slit experiment, investigations of which have continued along multiple lines for over a century as well, following multiple interpretations of QM, such as decoherence, consistent-histories, many worlds, or still others (with different versions within each type) or alternative theories (such as Bohmian mechanics or spontaneous collapse theories). These alternatives are beyond my scope, as are subtler versions of the experiment, such the delayed choice experiment or quantum eraser, discussed along RWR lines in [16] (pp. 65–76). To mention instructive recent treatments, with new experimental angles, are [1,2,17,18] and, along Bohmian lines [19]. As stated from the outset, however, I have not encountered any treatment of the double-slit experiment akin to the one pursued here in an extensive (even if not exhaustive, which would be impossible) perusal of literature on the experiment, from the works of founding figures, including Bohr, to recent sources.

As are concepts of reality in realist theories, the concept of reality without realism, RWR, is based on more general concepts of reality and existence, assumed here to be primitive concepts and are not given analytical definitions. By “reality” I refer to that which is assumed to exist, without making any claims concerning the *character* of this existence, claims that define realism. Realist physical theories or realist interpretations aim to offer at least a conception, but more commonly a representation of this reality, usually in terms of the objects considered by a theory. They also aim to predict the future course of this reality by means of this representation, either ideally exactly, deterministically, as in classical mechanics of individual or simple systems or relativity, or probabilistically, as in classical statistical physics or chaos theory. On the other hand, the absence of such claims allows one to place the reality considered or a stratum of this reality beyond representation or even conception. This placement defines this reality or this stratum as a “reality without realism,” RWR, and the corresponding interpretation of QM, or QFT, as an RWR interpretation. Thus, the word “reality” in “reality without realism” does not assume a *concept* of reality. It assumes the impossibility of a concept corresponding to this word.

I understand existence as a capacity to have effects on the world. The world may be defined, following L. Wittgenstein, as “all that is the case, …the totality of facts, not of things,” and, thus, is real in our experience, individual or shared [20] (p. 24). The universe will be understood as “all that is the case, … the totality of facts” in physics. A rigorous inference that something exists can only be made on the basis of its effects. RWR interpretations allow for representations of such effects as manifested in quantum phenomena, but not a representation or even a conception of how these effects come about. RWR interpretations do not assume a unified character of this reality, only manifesting itself differently in each experiment. This assumption is incompatible at least with strong versions of RWR interpretations, which preclude any conception of this reality and, hence, that of its unity. While each time unthinkable, an RWR-type reality is each time unique, manifesting its uniqueness in each encounter with this reality as an effect, each time unique in turn.

The assumption of the independent existence of nature or matter essentially amounts to the assumption that it existed before we existed and will continue to exist when we will no longer exist. This assumption has been challenged to the point of denying that there is any material reality. Plato and Bishop Berkeley are the most famous cases, respectively, ancient and modern, of this denial. It is true that any conception of how anything exists, or even that it exists, including as beyond thought, still belongs to thought. It does not follow, however, that something beyond thought does not exist. That we cannot imagine an entity that is neither continuous nor discrete, or even neither material nor mental, does not mean that such an entity does not exist in nature. The concept of RWR allows for such a possibility. RWR interpretations assume, by the Heisenberg postulate, that the ultimate reality responsible for quantum phenomena is beyond representation, which defines weak RWR interpretations, or, in strong RWR interpretations, beyond conception, making it unthinkable. This article adopts a strong RWR interpretation. (Unless qualified, by RWR interpretations, I shall hereafter refer to strong ones). Quantum phenomena themselves are, by contrast, available to our thinking and even to our immediate perception.

There is still the question of whether our inability to conceive of the ultimate reality responsible for quantum phenomena is related to one or the other of the following two possibilities. (A): This inability characterizes the situation in quantum physics *as things stand now*, while allowing that quantum phenomena or whatever may replace them will, at some point, no longer make this assumption and thus no longer make RWR-type interpretations viable, thus reverting to a realist view. (B): This inability reflects the *possibility* that this reality *will never become available* to thought. Logically, once (A) is the case, (B) is possible. There does not appear to be any experimental data compelling one to prefer either (A) or (B). (A) and (B) are, however, different in defining how far our mind can, in principle, reach in understanding nature. The strong RWR view is not about replacing what is unthinkable with new creations of thinking. Doing so, or replacing what is unknown with new knowledge, is an important aspect of mathematical and scientific thinking, including when the RWR view is adopted. The strong RWR view, as such, is, however, about creating new forms of thought, while assuming that there is something in nature or mind (possibly in mathematics [21]) that is beyond the reach of thought and may never be reached by it. This is the main reason to see (B) as possible. The qualification “as things stand now” applies, however, to (B) as well, although it might appear otherwise given that this view precludes any conception of the ultimate reality responsible for quantum phenomena not only now but also ever. It still applies because QT may, at some point, be given a definitive realist interpretation or replaced by an alternative theory that requires a realist interpretation. Either eventuality would make (B) obsolete even for those who held it in favor of a realist view of quantum physics, without, however, abolishing the concept of RWR itself, which may be applicable elsewhere.

The present interpretation also assumes the Bohr postulate, so named because Bohr strongly advocated it in his argumentation concerning quantum physics. According to this postulate, quantum phenomena and the observable part of the instruments used to establish quantum phenomena are described by classical physics, with the addition of special relativity in high-energy quantum regimes. The Bohr postulate is not required for an RWR interpretation, but is important for the present one, including in considering the double-slit experiment. Quantum phenomena are assumed to be defined by the fact that in considering the data found in them, the Planck constant, *h*, must be taken into account. I put aside possible qualifications of this definition (e.g., [7] (pp. 37–38) [22] (pp. 52–56). They are not germane for this article, because all quantum measurements considered here involve *h*, which is essential to the ultimate constitution of nature, also in reflecting the Planck scale (10^19^ GeV), the ultimate scale of this constitution in the present understanding of fundamental physics. In the present view, *h*, or any fundamental physical constant, such as *c*, belongs to this understanding, rather than being a property of nature itself. The fact, however, that *h* is experimentally based tells us that it is the effect of our interaction with nature by means of experimental technology and thus depends on something in nature.

For the reasons explained below, the present interpretation adds yet another postulate, the Dirac postulate, according to which the concept of quantum object is only applicable at the time of observation. For clarity, I state all three postulates:

*The Heisenberg postulate*, defining RWR interpretations, states that the reality ultimately responsible for quantum phenomena is beyond representation or (which is the form of the postulate assumed here) even conception.

*The Bohr postulate* states that observations and measurements used in quantum physics are represented by classical physics (cum special relativity in high-energy regimes), thus giving classical physics the fundamental role in quantum physics.

*The Dirac postulate* states that the concept of a quantum object is only applicable at the time of observation, and not independently.

These postulates are interpretive *assumptions* that could be falsified, although a falsification of an interpretation is more complex than a falsification of a theory by experimental evidence. The latter is not at stake in this article, which assumes both QM and QFT to be correct, as things stand now.

By the Heisenberg postulate, how quantum phenomena are possible is not represented by QM or QFT, but only predicted by it, in general probabilistically. It follows that, in RWR interpretations, QM or QFT has no physical connection, apart from these predictions, to either the ultimate nature of reality responsible for quantum phenomena or, if one assumes the Bohr postulate, to quantum phenomena, described by classical physics. Accordingly, in these interpretations, why the mathematics of QM or QFT correctly predicts the outcomes of quantum experiments is beyond knowledge or even conception. We know *how* this mathematics works, but we do not know and perhaps cannot know, or even conceive of, *why* it works. The situation is different if one assumes that the formalism of QM represents both the independent behavior of quantum systems and the transition to the classical outcomes of observation, for example, on lines of decoherence or consistent histories interpretations, which are realist. These approaches (also used in explaining the double-slit experiment) and challenges to them will be put aside.

In classical physics or relativity, as realist theories, the mathematical formalism *represents*, as a mathematized idealization, the physical reality responsible for the phenomena considered and connects these phenomena by continuous and classically causal processes. (The concept of classical causality is defined below.) Moreover, the phenomena and objects considered there can be identified with each other, for all practical purposes. The difference between objects and phenomena is still in place there, in accord with I. Kant’s view of objects as things-in-themselves vs. phenomena as appearances to our mind [23]. That means that one still ultimately deals with phenomena and not objects. The fact, however, that one can neglect or control the interference of observational devices into the course of the phenomena allows one to treat objects and phenomena as the same and the behavior of objects as independent of observation for all practical purposes.

This identification is no longer possible in considering quantum phenomena, in the constitution of which the role of observational instruments, precluding this identification, is irreducible, regardless of interpretation. Establishing quantum phenomena requires special instruments. Human bodies are sufficient in some cases and are models for the instruments used in classical physics, which instruments similarly do not disturb what is observed. This is not the case in quantum physics. Relativity also requires instruments, rods and clocks, beyond our bodies, because relativistic effects cannot be perceived by human bodies. This dependence, however, allows relativity to be a classically causal (in fact, deterministic) and, in the first place, realist theory, because one can observe the systems considered without disturbing them. Nobody has ever seen an ontologically quantum object, such as an electron or photon, or a macroscopic ontological quantum object, such as a Josephson junction. It is only possible to observe traces (such as spots on photographic screens or clicks in detectors) of their interactions with measuring instruments. To observe the quantum character of a Josephson junction, one needs a special instrument, in which and only in which quantum effects manifesting this character could be observed. Otherwise, one only sees two superconductors standing in a lab. In some cases of larger quantum objects, such as fullerene C60 molecules, it is possible to observe quantum diffraction or interference, including in the double-slit experiment [24].

The irreducibly intervening role of observational instruments in the constitution of quantum phenomena cannot be either neglected or controlled to enable one physically to reach the ultimate reality responsible for quantum phenomena, regardless of interpretation. As Bohr stated his 1927 article, “the Como lecture,” which outlined his first interpretation of QM, in classical physics or relativity “our … description of physical phenomena [is] based on the *idea* that the phenomena concerned may be observed *without disturbing them appreciably*” [13] (v. 1, p. 53; emphasis added). This lack of disturbance enables one to identify these phenomena with the objects considered, for all practical purposes. By contrast, “any observation of atomic phenomena will involve an *interaction [of the object under investigation] with the agency of observation* not to be neglected” [13] (v. 1, p. 54; emphasis added). One should note the subtle nature of this contrast: the interaction between the object under investigation and the agency of observation *gives rise* to a quantum phenomenon rather than *disturbs* it [13] (v. 2, p. 64). Relativity represented a step in this direction, insofar as, in contrast to Newtonian mechanics, space and time were no longer seen as preexisting (absolute) entities then measured by rods and clocks. They were *defined* by rods and clocks in each reference frame. Still, the intervention of observational instruments in the behavior of the objects considered could be disregarded, thus allowing the identification of these objects with observed phenomena and considering them independently of observation. This is no longer possible in considering quantum phenomena, regardless of interpretation, and hence also in realist interpretations of QM, or alternative theories, such as Bohmian mechanics. As noted, even in classical physics or relativity, this is only possible for all practical purposes, because all phenomena considered are still created by thought [7] (pp. vii–xxiv). Bohr is, accordingly, right to speak of “the idea,” and thus the assumption, “that the phenomena concerned [there] may be observed without disturbing them appreciably,” rather than saying that this is in fact the case. The constitutive role of experimental technology in defining relativistic and, more radically, quantum phenomena still allows for realist interpretations not only of relativity, where they are common, but also of QM or QFT, where they tend to be debated. On the other hand, this role opens the possibility of RWR interpretations of QM or QFT.

This role also entails an unavoidable discrimination, in each experimental arrangement, between quantum objects and instruments, and hence observed phenomena. This discrimination, according to Bohr, “may indeed be said to form a *principal distinction between classical and quantum-mechanical description of physical phenomena*” [25] (p. 701). This statement need not, and in Bohr’s understanding of it, does not, mean that QM describes these phenomena: in RWR interpretations, it does not, by the Heisenberg postulate. QM only, probabilistically, predicts them, while, as phenomena, they are physically described by classical physics, by the Bohr postulate. By “the quantum-mechanical description,” Bohr refers to the overall structure of QT in an RWR interpretation.

Both postulates were assumed by Bohr by the time of this statement in 1935, although still within a weak RWR interpretation, adopted in his reply to EPR, which I cite at the moment. His ultimate, strong RWR interpretation was introduced two years later. Regardless of interpretation, however, this discrimination precludes the identification of quantum objects and quantum phenomena, possibly, for all practical purposes, in the case of phenomena and objects considered in classical physics or relativity, different, on Kantian lines, as they may ultimately be. By the same token, as Bohr noted on the same occasion, while present in classical physics or relativity, the distinction between the objects considered and the instruments used “does not entail any difference in the character of the description of the phenomena concerned.” In quantum physics, where this distinction is of “fundamental importance,” it has “its root *in the indispensable use of classical concepts in the interpretation of all proper measurements*, even though the classical theories do not suffice in accounting for the new types of regularities with which we are concerned in atomic physics” [25] (p. 701). The latter is a crucial qualification: one needs a QT, such as QM or QFT, to account for these regularities, which are effects observed in measuring instruments and predicted by QM or QFT, without, in RWR interpretations, representing or even allowing one to conceive how these phenomena or these predictions are possible. These predictions are and, in these interpretations, can only be probabilistic, which is, as noted, fully in accord with what is observed, because identically prepared quantum experiments (whose preparation is possible because the observable parts of these instruments are described classically) in general lead to different outcomes.

Quantum predictions by QM or QFT require rules added to the formalism, rather than being contained in it, such as Born’s rule, which relates complex quantities of the formalism to real numbers corresponding to the probabilities of quantum events. While there are other rules that have the same function, I shall only refer here to Born’s rule, the first and still most commonly used. (There are claims for deriving such rules from the formalism of QM or, in my view, more plausibly, from other assumptions, which is, however, a separate subject.) I restate Born’s rule, in conjunction with RWR interpretations, under the assumption of the Bohr postulate. Mathematically, one takes the square moduli of the eigenvalues of the operators associated with quantum variables, such as position, momentum, or energy, or equivalently, multiplies these eigenvalues by their complex conjugates. Doing so gives one real numbers, corresponding, suitably normalized, to the probability densities of observable events. Born’s rule is a purely mathematical procedure, which has no representational relation to physical reality any more than does the formalism itself. The only such relations are those to quantum phenomena observed in measuring instruments and represented (along with the observable parts of these instruments), by the Bohr postulate, by classical physics, defined over R, and not that of QM, defined over C.

Born’s rule brings physics into the purely abstract mathematics of QM or QFT, including breaking the continuity of the formalism and making it relate to discontinuous quantum events defined by observation. Originally Schrödinger intended his wave function to represent, in wave terms, the ultimate reality responsible for quantum phenomena. His hope of achieving this goal became more difficult and was abandoned after he introduced his time-dependent equation, which required a complex wave function, initially defined as a real entity [7] (pp. 145–166). Attempts at realist interpretations of the wave function have never stopped, and Schrödinger, too, returned to such an attempt in the 1950s [26]. We do not know why Born’s or similar rules work. But then, we do not know why QM or QFT works either, insofar as, in RWR interpretations, it does not represent either the ultimate reality responsible for quantum phenomena or quantum phenomena themselves, represented by classical physics, by the Bohr postulate.

The Bohr postulate reflects the transition from the classical level of observation to the ultimate reality responsible for quantum phenomena by the initial preparation and back, from this ultimate reality to the classical level of observation in a subsequent experiment, testing a possible prediction on the basis of this preparation. According to Bohr,

The essential lesson of the analysis of measurements in quantum theory is thus the emphasis on the necessity, in the account of the phenomena, of taking the whole experimental arrangement into consideration, in complete conformity with the fact that all unambiguous interpretation of the quantum mechanical formalism involves the fixation of the external conditions, defining the initial state of the atomic system concerned and the character of the possible predictions as regards subsequent observable properties of that system. Any measurement in quantum theory can in fact only refer either to a fixation of the initial state or to the test of such predictions, and it is the combination of measurements of both kinds which constitutes a well-defined phenomenon [experiment?].

[27] (p. 101)

I would replace “phenomenon” with “experiment” because, given Bohr’s definition of the concept of phenomenon, discussed below, as referring strictly to *an event that has already taken place*, rather than to a possible event, one deals with two phenomena corresponding to two events considered. One must specify two measurements and arrangements: the first is the actual measurement or phenomenon, and the second is a possible future measurement or phenomenon that would enable one to verify one’s probabilistic or statistical predictions, after repeating the experiments many times. One begins an experiment by classically preparing the instrument and registering, at time *t*_1_, the data obtained by the interaction between this instrument and a quantum object. Doing so sets up the workings of the ultimate reality considered, placed beyond representation or even conception, in RWR interpretations. This measurement is a “classical” to “quantum” transition: from the classical observation to the ultimate reality responsible for quantum phenomena. Such data themselves belong to observed quantum phenomena and are independent of any theory. One, however, needs a theory, such as QM, to predict (by using these data) the data obtainable in future experiments.

One can, for example, use Schrödinger’s time-dependent equation (considering it in one dimension for simplicity)

(1) $$i\hbar \frac{\partial \psi (x,t)}{\partial t}=\left[-\frac{\hbar^{2}}{2m}\frac{\partial }{\partial x^{2}}+V(x,t)\right]\psi (x,t).$$

for making a prediction concerning a future coordinate measurement associated with an electron on the basis of a previously performed position measurement, at time *t*_1_ [28] (p. 74). Here *m* is the mass of the particle, *V*(*x*,*t*) is the potential that represents (as an operator) the environment of the electron, as part of the initial conditions, and ψ (x, t) is the wave function that assigns a complex quantity to each point *x* and each time *t*. The wave function is associated with the probability amplitude, by providing, via Born’s rule, the probability density by the square modulus of |$\left.\psi \right\rangle$, ${\left\|\psi \right\|}^{2}$, which is positive and can be normalized so that this probability is between zero and one. Then, to confirm such a prediction, one sets up a suitable observational device and makes a new observation, registering an outcome of the experiment, thus predicted, at time *t*_2_. The instrument needs to be prepared in accordance with this prediction for the coordinate measurement. This is because one can always perform a different type of measurement, say, that of the momentum, at *t*_2_, which will irrevocably disable verifying the prediction concerning *q*. This is the transition from “quantum” to “classical” reality in this experiment. One cannot associate any concept of “quantum” with the ultimate reality responsible for quantum phenomena in RWR interpretations. I speak of this reality as “quantum” because classical physics, which describes the data observed, cannot predict it. Mathematically, the classical to “quantum” transition means putting the data, say, the measured value of the coordinate and time *t*_1_, into Schrödinger’s equation. One can then, adding Born’s rule to the solution, predict the probability of the quantum to classical transition, for finding the value of *q* with a given interval a ≤q ≤ b. The quantum to classical transition is the measurement that confirms this prediction, which confirmation cannot be guaranteed.

**Figure 1:**
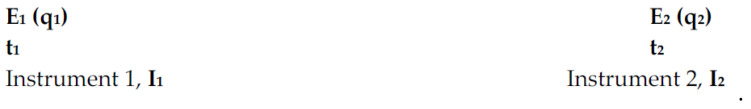
*Experiment*: Both instruments are prepared for measuring **q**. *Theory*: QM, by using **q_1_** in its equations, cum Born’s rule, predicts the probability or (if the experiment is repeated many times) the statistics of finding **q_2_** in the interval **[a, b]**, **a** ≤ **q_1_** ≤ **b**.

The situation and the overall argumentation outlined in this section (apart from the Dirac postulate, discussed below), can be mapped by the following diagram (Figure 1) representing two events, **E_1_** and **E_2_**, relating to two positions measurements, **M_1_** and **M_2_**, of **q_1_** and **q_2_**, in one dimension, where **M_1_** is prepared and made, and **M_2_** is predicted for **a** $\leq q_{2}$ ≤ **b** and then made to test this prediction. (I still consider a one-dimensional case.)

The diagram reflects the fundamental difference between classical physics or relativity and quantum physics by virtue of the irreducible role of observational instruments in establishing quantum phenomena. In classical physics or relativity, the intervention of measuring instruments could be neglected or controlled, allowing one, in dealing with individual or simple systems, to identify the observed phenomenon with the object considered. The corresponding diagram would be changed from the one above by removing measuring devices from it, as merely auxiliary. In quantum physics, this intervention cannot be neglected or controlled, which makes the phenomena observed in observational instruments irreducibly different from the quantum objects considered. As stated, nobody has ever seen an actual electron or photon, or any quantum object, as such. One can only observe traces of their interactions with measuring instruments. I indicate this by leaving the space between **I_1_** and **I_2_** empty, as Bohr does in his diagrams illustrating quantum experiments and observational devices used in them (e.g., [13] (v. 2, pp. 48–55). RWR interpretations respond to this experimental situation. In these interpretations, no representation of the transition between **E_1_/M_1_** and **E_2_/M_2_**, or even no conception of how this transition happens, is possible. It is, however, possible to predict transition probabilities between these events. To do so, one does need a theory, such as QM, or in high-energy regimes, QFT, which would predict these probabilities or statistics. Quantum phenomena and the data observed in them are represented by classical physics, by the Bohr postulate.

Two key concepts defining classical physics and relativity, measurement and classical causality, become no longer applicable in QT in RWR interpretations. The classical concept of measurement belongs to classical physics and the history that shaped it, beginning with ancient Greece and the rise in geometry, geo-*metry*, arguably the first science of measurement. In the present view, a quantum measurement *does not measure*, or in the first place, *is not an observation of*, any property that the ultimate reality responsible for quantum phenomena would be assumed to possess before or even during the act of observation. The concept of observation requires redefinition as well. An act of observation is a unique event of creation that establishes, *creates*, a quantum phenomenon by an *interaction* between the instrument and a quantum object. Then the data observed can be measured classically, just as one measures what is observed in classical physics or relativity. There, however, what is observed or measured could be associated with the object considered, with the difference between observation and measurement having no physical significance. In quantum physics, in RWR interpretations, the difference between observations, which construct phenomena, and measurements, which classically measure properties of physical entities (classical objects) represented by phenomena, is fundamental.

This view gives a central significance to the category of event, as defining a new physical situation each time, while making any preceding event no longer meaningful for any predictions from this point on. QT becomes a theory of transition probabilities between events and phenomena created, defined by experimental technology, and our decisions concerning which experiment to perform. The concept of event assumed here, especially (a) as an effect of an RWR-type of reality observed in experimental technology and (b) as the creation of a physical phenomenon, defined classically, makes RWR interpretations (if they assume the Bohr postulate), different from other event-based interpretations of QM or QFT, especially realist ones (as most of these interpretations are), such as C. Rovelli’s relational interpretation [29] or that of R. Haag [30], or those developed in more recent approaches (e.g., [31] and references there). The Bohr postulate also makes such interpretations different from other interpretations centered on transition probabilities between events, while assuming that all systems considered in quantum physics, for example, “the friend” in Wigner’s friend experiment, are quantum. This assumption is found, for example, in Quantum Bayesianism (QBism), based on the role of human agents in predicting such probabilities [32,33]. Indeed, QBism claims not only the subjective nature of these predictions, a view close to the one assumed here, but also of the outcomes of quantum observations and measurements, a view not assumed here. In the present view, following Bohr, these outcomes or, more precisely, the ways of representing them, are “objective.” They are, however, understood as “objective” only in the sense of being unambiguously definable and communicable, rather than in the realist sense of representing the “objects” considered (here the ultimate reality responsible for quantum phenomena), which is precluded in RWR interpretations. This sense of objectivity allows for the subjective or (our experiences are rarely, if ever, entirely subjective) personal character of human agents’ experiences of the outcomes of quantum experiments.

The nature of causality in QM or QFT changes as well in RWR interpretations, which makes classical causality no longer applicable [7] (pp. 207–218). By “classical causality” I refer to the claim that the state, *X*, of a physical system is determined, in accordance with a law, at all future moments of time once its state, *A*, is determined at a given moment of time, and state *A* is determined by the same law by any of the system’s previous states. This assumption implies a concept of reality, which defines this law, thus making this concept of causality ontological. The main reasons for my use of the term “classical causality,” rather than just causality, commonly used to designate this type of concept, are that it is possible to introduce alternative, probabilistic, concepts of causality, applicable in QM, including in RWR interpretations, where classical causality does not apply. I shall explain this type of concept below. Some, beginning with P. S. Laplace, have used “determinism” to designate classical causality. I use “determinism” as an epistemological category referring to the possibility of predicting the outcomes of classically causal processes ideally exactly. In classical mechanics, when dealing with individual or small systems, both concepts are coextensive. On the other hand, classical statistical mechanics or chaos theory are classically causal but not deterministic in view of the complexity of the systems considered, which limits us to probabilistic or statistical predictions concerning them.

As stated from the outset, in dealing with quantum phenomena, deterministic predictions are not possible even in considering the most elementary systems. This is because the repetition of identically prepared quantum experiments (whose preparation is possible because the data observed are described and controlled classically) in general leads to different recorded data, and unlike in classical physics, this difference cannot be diminished beyond the limit, defined by *h*, by improving the capacity of our measuring instruments. On the other hand, their interaction with quantum objects cannot be controlled, compelling Bohr to speak of “the finite and uncontrollable interaction between the objects and the measuring instruments in the field of quantum theory” [25] (p. 700). It follows that the probabilistic or statistical character of quantum predictions must hold in interpretations of QM or theories of quantum phenomena (such as Bohmian mechanics) that are classically causal. QM or QFT, in RWR interpretations, are not classically causal because the ultimate reality responsible for quantum phenomena is assumed to be beyond a representation or even conception. Classical causality would imply at least a partial conception of this reality. This situation implies a different reason for the recourse to probability or statistics in QT in RWR interpretations, as often emphasized by Bohr. Thus, he said the following:

[I]t is most important to realize that the recourse to probability laws under such circumstances is essentially different in aim from the familiar application of statistical considerations as practical means of accounting for the properties of mechanical systems of great structural complexity. In fact, in quantum physics we are presented not with intricacies of this kind, but with the inability of the classical frame of concepts to comprise the peculiar feature of indivisibility, or “individuality,” characterizing the elementary processes.

[13] (v. 2, p. 34)

The “indivisibility” refers to the indivisibility of phenomena in Bohr’s sense, as the impossibility of considering quantum objects independently of their interactions with these instruments. “Individuality” refers to the fact that, as a unique creation, each phenomenon is individual and unrepeatable, as well as discrete relative to any other phenomenon. This individuality also embodies the essential randomness of quantum phenomena, not found in classical physics, because even when one uses probability there, at bottom one still deals with individual processes that are classically causal. Importantly, quantum physics only *contains* an essential randomness, rather than being entirely random, because it allows for probabilistic or statistical predictions (purely random events do not) and, more crucially, correlations, such as EPR-type correlations, at stake in Bell’s or the Kochen–Specker theorem, or those of the interference pattern in the double-slit experiment. As noted, one of the greatest mysteries of quantum physics, perhaps its greatest mystery, is how random individual events can, under certain circumstances, give rise to an order, a statistical correlational order, but an order nonetheless [7] (pp. 253–256). QM predicts these correlations, but in RWR interpretations, it does not explain them any more than it explains how any single outcome involved comes about. (I put aside the question of statistical vs. probabilistic interpretations of QM, considered from an RWR perspective in [34] (pp. 173–186).) One can still speak of causality, “quantum causality,” in the RWR interpretation. This causality is probabilistic and is defined as follows: “An *actual* event that has happened determines which events *may or (in view of complementarity) may not* happen and be predicted with *one probability or another*, which is not the same that any of them *will* happen. This event, A, at time *t*_0_ defines certain *possible*, but *only possible* future events, say, X, at time *t*_1_” [7] (pp. 208–209). A full discussion of this concept is found in [7] (pp. 207–218).

In grounding his ultimate RWR interpretation, Bohr adopted the term “phenomenon” to refer strictly to what is observed, *classically*, in measuring instruments:

I advocated the application of the word phenomenon exclusively to refer to *the observations obtained under specified circumstances*, including an account of the whole experimental arrangement. In such terminology, the observational problem is free of any special intricacy since, in actual experiments, all observations are expressed by unambiguous statements referring, for instance, to the registration of the point at which an electron arrives at a photographic plate. Moreover, speaking in such a way is just suited to emphasize that the appropriate physical interpretation of the symbolic quantum-mechanical formalism amounts only to predictions, of determinate or statistical character, pertaining to individual phenomena appearing under conditions defined by classical physical concepts [describing the observable parts of measuring instruments].

[13] (v. 2, p. 64; emphasis added).

The difference between phenomena and objects has its genealogy in Kant’s philosophy, which initiated the modern history of this difference, conceived by Kant more radically than previously, from the ancient Greeks on. Kant distinguishes between objects as things-in-themselves, as existing independently of our phenomenal perception, and phenomena, as entities created by our mind, which may not correspond to the things-in-themselves as objects in nature or possibly the mind that they aim to represent [23]. In RWR interpretations, quantum phenomena are not related to quantum objects at all, but if one assumes the Bohr postulate, they represent classical physical objects observed in quantum instruments, such as a spot on a photographic plate. RWR interpretations, especially strong ones, are also more radical than Kant’s system. Things-in-themselves were assumed by Kant to be beyond knowledge but not beyond conception, at least a *hypothetical* conception, possibly justified only practically by its applications [23] (p. 115). In strong RWR interpretations, what is practically justified is not a possible conception of the ultimate reality responsible for quantum phenomena, but the impossibility of such a conception [7] (pp. 57–58).

As defined by “*the observations* [already] *obtained under specified circumstances*,” phenomena only refer to events that have already occurred and not to possible future events, such as those predicted by QM. This is the case even if these predictions are ideally exact, which they can be in certain circumstances, such as those of EPR-type experiments. The reason that such predictions cannot define a quantum phenomenon is as follows. A prediction for variable *Q* (for example, that related to a coordinate, *q*) cannot, in general, be assumed to be confirmable by a future measurement in the way it can be in classical physics or relativity, where all measurable quantities can, in principle, always be defined simultaneously and assume to have a reality independent of measurement. In quantum physics this is not the case. One can always, instead of the predicted measurement, perform a complementary measurement, that of *p* (the momentum), which will leave any value predicted by using *Q* undetermined by the uncertainty relations. This measurement would irrevocably preclude associating a physical reality corresponding to a coordinate *q* when one measures *p* [7] (pp. 210–212). (This point is crucial for understanding the EPR experiment and countering EPR’s argument [35] along the lines of Bohr’s reply [7] (pp. 227–257)). One can never speak of both variables, when considered simultaneously, unambiguously, even if they are associated with measuring instruments. Any reference, even that to a single property of a quantum object, is impossible in RWR interpretations even at the time of observation, which places a quantum object subject beyond conception even then. In a quantum experiment one always deals with a system comprising an object and an instrument. There is, on the one hand, always discrimination between an object and an instrument, and, on the other, the impossibility of separating them. This impossibility compelled Bohr to speak, in defining his RWR interpretation, of “the essential ambiguity involved in a reference to physical attributes of objects when dealing with phenomena where no sharp distinction can be made between the behavior of the objects themselves and their interaction with the measuring instruments” [13] (v. 2, p. 61).

The present interpretation adds the Dirac postulate to the Heisenberg and Bohr postulates. The postulate states that the concept of a quantum object is applicable, still as an inconceivable (RWR) entity, only at the time of observation and not independently. While Bohr’s argumentation might be seen as suggesting the Dirac postulate, Bohr never stated this type of postulate and appears to have seen (ontologically) quantum objects as existing independently, as RWR entities. Unlike the Heisenberg postulate by Heisenberg and Bohr postulate by Bohr (without using these designations), this postulate was not considered by Dirac. My designation “Dirac postulate” originates in Dirac’s discovery of antimatter as a consequence of Dirac’s equation for the (free) relativistic electron [36] (p. 255):

(2) $$\left(\beta mc^{2}+\sum_{k=1}^{3}\alpha_{k}p_{k}c\right)\psi \left(x,t\right)=i\hbar \frac{\partial \psi \left(x,t\right)}{\partial t}\;\;$$

$$\alpha_{i}^{2}=\beta^{2}=I_{4}$$

(*I*_4_ is the identity matrix)

$$\alpha_{i}\beta +\beta \alpha_{i}=0$$

$$\alpha_{i}\alpha_{j}+\alpha_{j}\alpha_{i}=0$$

$\psi \;\left(x,\;t\right)$ is the wave function for the electron with rest mass *m*. *p*_1_, *p*_2_, and *p*_3_ are the components of the momentum, the operator akin to that used in Schrödinger’s equation in QM. The new elements in Dirac’s equation are the four 4 × 4 matrices *α*_1_, *α*_2_, *α*_3_, and *β*, and the four-component *ψ*, necessary because the evolution of *ψ* at any given point in configuration space is a “bispinor.” The name is due to the fact that *ψ* is a superposition of Hilbert-space vectors that allow one to predict a spin-up electron or a spin-down electron, or a spin-up positron or a spin-down positron. Dirac’s equation contains spin automatically, in contrast to QM, where the spin is handled separately by using Pauli (2 × 2) matrices. The wave function *ψ (t*, ***x**)* takes value in a Hilbert space *X* = C^4^ (Dirac’s spinors are elements of *X*). For each *t*, *y (t*, ***x**)* is an element of *H* = L^2^ (R^3^) ⊗ *X*. 

While originally designed for an electron, the equation revealed itself to be an equation for both the electron and the positron. It reflected and, in fact, led to the discovery that the identity of a particle-type could no longer be assumed in successive observations, as in classical physics or even nonrelativistic QT: the initial observation can register an electron, while the next one a positron, or a photon, or an electron-positron pair, with the probabilities defined by the same equation. Once one moves to still higher energies, the panoply of possible outcomes becomes greater. In QED, one only has electrons, positrons, and photons; in QFT, depending on how high the energy is, one can literally find any known and possibly, as some hope, as yet unknown elementary particle or combination, that is, the corresponding effects will be registered. The situation is further complicated by the role of virtual particles, the existence of which is under debate [7] (pp. 273–306).

This situation makes adopting the Dirac postulate nearly imperative in QFT. There are, however, reasons to use it in RWR interpretations of QM. According to Heisenberg,

There is no description of what *happens* to the system between the initial observation and the next measurement. … The demand to “describe what happens” in the quantum-theoretical process between two successive observations is a contradiction in adjecto, since the word “describe” refers to the use of classical concepts, while these concepts cannot be applied in the space between the observations; they can only be applied at the points of observation. … [T]he problems of language are really serious. We wish to speak in some way about the structure of the atoms [as made of particles] and not only about ‘facts’—the latter being, for instance, the black spots on a photographic plate or the water droplets in a cloud chamber. However, we cannot speak about the atoms in ordinary language.

[37] (pp. 57, 145, 178–179).

Nor is it possible in terms of ordinary concepts, from which ordinary language is indissociable. One could, in principle, use mathematical concepts in representing physical reality, apart from an ordinary-language description of it, even if it is difficult in practice to define these concepts without using ordinary language. Heisenberg’s formulation does allow for a *mathematical representation* of the reality between observations, defining quantum phenomena. Indeed, this became the view adopted by Heisenberg at the time of this statement [37] (pp. 59–75, 145, 167–186), as dicussed in [7] (pp. 75–76). Heisenberg no longer assumed the Heisenberg postulate, at least not in the form used by him in his discovery of QM. His view shifted to a form of mathematical realism, akin to structural realism [38]. Heisenberg still assumed, in accord with Bohr’s view, the Bohr postulate [7] (pp. 207–218). In Bohr’s view, however, in its ultimate strong RWR form, or in the present view, this reality is an RWR-type reality and, as beyond all conception, is also beyond any mathematical conception, let alone mathematical representation. For the moment, if “there is no description [let alone, conception] of what *happens* to the system between the initial observation and the next measurement,” the Dirac postulate becomes a reasonable assumption even in low-energy quantum regimes. It is, as will be seen, also suggested by the double-slit experiment in the present interpretation, especially by the role of entanglement there.

In the present interpretation, an ontologically quantum object, such as an electron or a photon, is a manifestation of the ultimate reality responsible for quantum phenomena at the time of the interaction between this reality and observational instruments, enabling quantum phenomena. Accordingly, while coextensive with (technically preceding) a quantum phenomenon, as the classically observed effect of this interaction, an ontologically quantum object is still beyond representation or conception. An experimentally quantum but physically classical object, such as, the diaphragm with two slits (D2) in the double-slit experiment, can be observed as a classical object during the same time, keeping in mind that it can only become an experimentally quantum object if coupled to an ontologically quantum object, as in the case of D2 in S2, one, O, passing through it.

In the case of ontologically quantum objects, some observed effects are variable and others are constant or invariant. Thus, in any quantum regime, two electrons could be distinguished by changeable properties *associated* with them, in RWR interpretations, again, strictly as effects observed in measuring instruments, such as their positions in space or time, momenta, energy, or the directions of spins. Such properties are not invariant and are subject to the uncertainty relations and complementarity. It is also possible to locate, by observations, two different electrons in separate regions in space. It is, however, not possible to distinguish electrons from each other on the basis of their mass, charge, or spin. Spin is a purely quantum variable and hence can only be associated with ontologically quantum objects; classical objects do not possess spin. These quantities are invariant and are not subject to the uncertainty relations or complementarity. As H. Weyl said, “the possibility that one of the identical twins Mike and Ike is in the quantum state E1 and the other in the quantum state E2 does not include two differentiable cases which are permuted on permuting Mike and Ike; it is impossible for either of these individuals to retain his identity so that one of them will always be able to say ‘I’m Mike’ and the other ‘I’m Ike.’ Even in principle one cannot demand an alibi of an electron!” [39] (p. 241). An experimentally quantum object that is ontologically classical has no strictly (only locally or temporarily) invariant physical properties, and most of its properties can be observed directly or, in any event, without observational instruments defining them.

The Dirac postulate allows one to maintain both the indistinguishability of particles of the same type and the strict distinguishability of the types themselves because both features can be consistently defined by the corresponding sets of effects manifested in measuring instruments. An elementary particle of a given type, say, again, an electron, is specified by a discrete set of possible phenomena or events (the same for all electrons), observable in measuring instruments in the experiments associated with particles of this type. An elementary particle can only exist as part of a composite system, consisting of this particle and a measuring instrument, which system has a registered effect upon the observable, classically describable, part of this instrument. The elementary character of a particle is defined by the fact that there is no experiment that allows one to associate the corresponding effects on measuring instruments with more elementary individual quantum objects. Once such an experiment becomes conceivable or is performed, the status of a quantum object as an elementary particle could be challenged or disproven, as happened when hadrons and mesons were discovered to be composed of quarks and gluons. If so, this composite nature will manifest itself in a new set of effects observed in the corresponding experiments. It follows that, as defined in terms of such effects, the present concept of an elementary particle does not imply that “elementary particles” are fundamental elementary constituents, “building blocks,” of nature. This assumption is precluded by RWR-type interpretations, as is any assumption concerning this constitution. Nor is it possible to apply to elementary particles any physical concept of a particle, any more than any other concept, such as wave or field, although the concept of a quantum field could be defined as a mode of RWR-type reality, rather than a quantum object [7] (pp. 273–306).

The following question might, however, be asked. Would assuming the Dirac postulate still allow one to speak of the same ontologically quantum object, say, again, the same electron, in two or more successive measurements? One might, for example, consider (speaking in more conventional terms) two position measurements in the double-slit experiment in the “which-way” setup, the first, M1, defined by a slit in D2, through which an electron is assumed to have passed as registered by a counter, and the second, M2, by a collision between it and the screen. Each measurement defines an electron with the same mass, charge, and spin (which could be measured simultaneously with either position measurement), in two different positions at two different moments in time. The case can be given an RWR interpretation insofar as all these properties (mass, charge, and position) are those of measuring devices, interacting with the electron rather than of this electron, placed beyond representation or conception even at the time of measurement, and not definable between measurements by the Dirac postulate. The question is as follows: Do these two measurements register the same electron? Rigorously, if the concept of quantum object is only applicable at the time of observation or measurement, M1, then a prediction based on a given measurement and the new measurement, M2, based on this prediction could only concern a new electron, and not the electron defined at the time of M1. To consider them as the same electron is, however, a permissible idealization in low-energy (QM) regimes, an idealization statistical in nature, because a collision with the screen is not guaranteed, although the probability that it will not happen is low. In practical terms, whether it is the same or a different electron has no effect on the event. First, the prediction concerning the position of the electron will be the same either way. Secondly, invariant properties will be the same without allowing us to distinguish electrons from each other individually. The Fermi-Dirac statistics for electrons as fermions (which have half-integer spin), linked to Pauli’s exclusion principle, will be different from Bose-Einstein statistics for bosons (which have integer spin), for which there is no exclusion principle. These properties and features are purely quantum and, hence, can only be associated with ontologically quantum objects and systems.

### 2.2. Complementarity: Decision and Reality Without Waves or Particles

This section discusses *Bohr’s concept* of complementarity, especially as it functions in his ultimate interpretation of quantum phenomena, where it applies to phenomena in Bohr’s sense. My emphasis on *Bohr’s concept* reflects the importance of understanding this concept as conceived by Bohr. The concept can be, and has been, defined otherwise, even when inspired by Bohr. It had undergone changes in Bohr’s work as well, correlative to those in Bohr’s interpretation, especially vis-à-vis Bohr’s initial definition of complementarity in the Como lecture. These changes were considered by this author previously, especially in [7]. As noted at the outset, there are alternative interpretations of Bohr’s interpretation and hence of complementarity or his other concepts and their development. A comprehensive collection of articles on Bohr’s thinking, including concerning complementarity, from diverse up-to-date philosophical perspectives, is assembled in [9].

As defined, arguably, most generally, complementarity is characterized by the following three features:

(A) A mutual exclusivity of certain phenomena, entities, or conceptions;

(B) The possibility of considering each one of them separately at any given point; 

(C) The necessity of considering all of them at different moments of time for a comprehensive account of the totality of phenomena that one must consider.

This formulation as such was not expressly stated by Bohr, who never offered a single definition of complementarity. I would argue, however, that it is consistent with Bohr’s understanding of complementarity, at least following the Como lecture, as is clear from his multiple explanations of the concept (e.g., [13], (v. 2. p. 40)). In any event, this is the definition of the concept to be adopted here, as a general definition, implying, insofar as it applies to Bohr’s concept, an *interpretation* of Bohr’s concept. In quantum physics, in Bohr and other RWR interpretations, complementarity implies two incompatible situations of what is observed, as phenomena, in the instruments used. The *possible* information about a quantum object, the information *to be found* only in observational instruments, is obtainable in a mutually exclusive way under incompatible experimental conditions (e.g., [13] (v. 2, p. 40)). By contrast, once made, either measurement, say, that of the position, will provide the *complete actual* information about the object considered, as complete as possible, at this moment in time. One could never obtain the complementary information, provided by the momentum measurement, at the same time, because to do so, one would need simultaneously to perform a complementarity experiment on it, which is impossible. On the other hand, one can, by (B), decide to perform either one or the other experiment. Each experiment establishes the only possible reality at this moment in time, and the alternative decision would establish a different reality. Accordingly, rather than selecting one or another part of a preexisting reality, as our experimental decisions do in classical physics or relativity, a decision which experiment to perform establishes (a) the *single* reality defined by the *type* of quantity observed and (b) a possible, probabilistically predictable future course of reality, while precluding the complementary alternative.

Thus, in the present understanding of the concept, including in Bohr’s work, complementarity is not only about the mutual exclusivity of certain entities but also about performing quantum experiments by human agents. One can set up a device to perform an experiment and treat a natural event as an experiment, but any such setup is only possible by a human agent, individual or collective, or by a device, such as a computer, which, at least as things stand now, must still be programmed by a human agent to do so. That one can freely or sufficiently freely make a decision (a preferable category to “choice”) concerning which experiment one wants to perform is, as Bohr noted, in accordance with the very idea of experimentation in all physics or science [25] (p. 699). Contrary to classical physics or relativity, however, in quantum physics, implementing one’s decision allows one to make only certain types of predictions and will irrevocably exclude certain other, *complementary*, types of possible predictions. (Other predictions than complementary remain possible simultaneously.) Either decision defines the reality considered and its possible (but only possible, given the irreducible probabilistic character of all future quantum events) future course in two alternative ways. There is only one such course in classical physics or relativity, even though it can sometimes only be known or predicted partially. By the same token, in these theories our decisions, even if they define new experimental situations, merely follow what would happen in any event, without interfering with the phenomena observed. They always allow us to determine, as independent of observation, all variables necessary for defining the future course of reality, in accord with classical causality. This is impossible in quantum physics because of the uncertainty relations and complementarity, even if one assumes the underlying classical causality, as in certain interpretations of QM (such as many-worlds ones), or in Bohmian mechanics, which contains the uncertainty relations and complementarity. In such cases, the situation is only practically, epistemologically different from classical physics or relativity, which do not contain complementarity (in Bohr’s sense). Ontologically, these interpretations or theories are analogous to classical physics and relativity, insofar as such experimental decisions only determine one or the other possible information concerning the same ultimate reality. The difference is that, in the absence of the uncertainty relations and complementarity, in classical physics or relativity, it is in principle possible, in considering individual or simple systems, to know all of the reality considered and predict its future course ideally exactly. This is never possible, even for the simplest quantum systems, in quantum physics, even in realist or classically causal interpretations or theories. In any event, in RWR interpretations, beginning with that of Bohr, it is not an *epistemological* matter of knowing one or the other complementarity part of the same reality. Once enacted, either of the two possible complementary decisions *ontologically* defines the only reality there is and its possible future course, and in principle excludes the complementary reality and its possible future course.

The role of decision is crucial in quantum physics and is one of the key differences between it and classical physics or relativity, which do not contain either the uncertainty relations or complementarity [7] (pp. 207–218) [40]. We always have at least relative freedom to make this decision or to change our decision, which capacity defines free will here. This freedom is relative because it may be limited by various factors (social, psychological, or others), which makes decision a preferable category to that of choice. Nor, given the *uncontrollable* nature, vis-à-vis in classical physics or relativity, of the interactions between quantum objects and measuring instruments, can one control the outcomes of quantum experiments [25] (p. 697). One can only control the behavior of observational instruments because they can, in their observable parts, be described classically. This gives quantum measurements a form of objectivity, insofar as one can unambiguously define and communicate their outcomes, while predictions entail a form of subjectivity, as personal assignments of probabilities of these outcomes. This combination differentiates the present (or Bohr’s view) from QBism, where, as explained above, the outcomes of measurements are considered as subjective as well [32,33]. This freedom is denied by so-called “superdeterminism,” according to which all our decisions are pre-determined in advance, from the Big Bang on. This view, controversial in any event, will be put aside as representing the extreme form of dogmatism in classically causal and deterministic thinking. The present RWR view of quantum physics may be seen as “superprobabilism,” as representing, thus far, the most radical departure from correlatively both realism and classical causality. In this view, again, the recourse to probability in QT is no longer a practical, epistemological matter, as in classical statistical physics or chaos theory, due to our lack of knowledge concerning the underlying behavior of the mechanically complex systems considered, while their elementary constituents could in principle be predicted exactly deterministically. Nor is it due to a disturbance of the classically causal behavior of individual or simple systems by observations, as in Bohmian mechanics. In RWR interpretations, this recourse arises because no knowledge or even conception concerning the behavior of quantum objects is possible, even the simplest possible ones.

Complementarity helps to establish the possibility of an unambiguous definition and communication of what can be said concerning quantum phenomena, while establishing the difference between quantum physics and classical physics or relativity. There, all our decisions can be defined in a single domain of the reality considered, where we make predictions, exact or probabilistic, in the same probability space. This is sometimes referred to as the “unicity” of this domain, which may be the Universe as a whole, as in Newton’s *Principia*, where the Universe is governed by absolute space and time, or in relativistic cosmological theories without these concepts. This unicity is represented by the equations used, such as Lagrangian equations. These equations, such as those in classical physics

(3) $$\frac{\partial L}{\partial q_{j}}-\frac{d}{dt}\;\frac{\partial L}{\partial {\dot{q}}_{j}}=0$$

enable the representations and predictions concerning all variables considered [41] (p. 36). Relativity only allows one to maintain this unicity locally, in general relativity by means of Einstein’s (Lagrangian) equations.

In QT, all domains considered are doubled by our decisions to establish one interrelated set of them or the other, without the possibility of unifying them. The equations of QM or QFT, such as Schrödinger’s equation, can only be written for one of the two (or more) complementary physical realities and in one mathematical “space,” a Hilbert space over C (rather than defined over R, as are the equations of classical physics and relativity), a key aspect of the difference between the mathematics of QT and that of classical physics or relativity. A measurement of the coordinate, at time *t*_1_, allows one to use one form of Schrödinger’s equation, that for the position variable, to predict the probability of a future position measurement within a given range at future time *t*_2_:

(4) $$i\hbar \frac{\partial }{\partial t}\psi \left(x,t\right)=\frac{1}{2m}{\left(-i\hbar \frac{\partial }{\partial x}\right)}^{2}\psi \left(x,t\right)+V\left(x,t\right)\psi \left(x,t\right).$$

A measurement of the momentum (in the same direction) at time *t*_1_ allows one to use a different form of Schrödinger’s equation, that for the momentum variable, to predict the probability of a future position measurement within a given range at future time *t*_2_:

(5) $$i\hbar \frac{\partial }{\partial t}\phi \left(p,t\right)=\frac{p^{2}}{2m}\phi \left(p,t\right)+V\left(i\hbar \frac{\partial }{\partial p},t\right)\phi \left(p,t\right)$$

These equations can be mathematically transformed into one another by using the Fourier transform:

(6) $$\phi \left(p,t\right)=\int dx\frac{e^{-i\frac{p}{\hbar }x}}{\sqrt{2\pi \hbar }}\psi \left(x,t\right)$$

This doubled structure ([28] (pp. 73, 105)) cannot be unified into a single physical domain considered or allow for a single equation or a single system of equations that would enable one to handle both variables at any moment in time, as is possible in classical physics or relativity. There can be compatible, rather than mutually exclusive, decisions concerning possible predictions (using commuting operators) associated with different variables. The spaces of these decisions are unified. A defining aspect of quantum physics is, however, that they cannot always be unified, and when they cannot be, they create incompatible realities, rather than describe different parts of the same reality. In Bohr’s words,

We are not dealing with an incomplete description characterized by the arbitrary picking out of different elements of physical reality at the cost of [sacrificing] other such elements, but with a rational discrimination between essentially different experimental arrangements and procedures which are suited either for an unambiguous use of the idea of space location, or for a legitimate application of the conservation theorem of momentum. Any remaining appearance of arbitrariness concerns merely our freedom of handling the measuring instruments, characteristic of the very idea of experiment.

[25] (p. 699).

From this perspective, the equations of QM, such as Schrödinger’s equation, or QFT, such as Dirac’s equation, are *not equations of motion*, a concept difficult to define in quantum physics and strictly inapplicable in RWR interpretations. They are *equations of decisions (concerning which experiment to perform) and transition probabilities between events*, observed in measuring instruments as information represented by classical physics, with the addition of special relativity in high-energy regimes.

The reader has probably noticed that my discussion of complementarity has not thus far mentioned wave-particle complementarity, with which the concept of complementarity is often associated. This is not an oversight on my part. As stated from the outset, in the present view, this complementarity is not only unnecessary, including in considering the double slit experiment, but is also misleading. This complementarity, I also contend, did not play a significant role in Bohr’s thinking either, again, in the present interpretation of his available writings (including correspondence), especially after the Como lecture, which introduced the concept of complementarity, defined differently than in his later works [13] (v. 1, pp. 54–55). Even there, while possibly assumed, wave-particle complementarity was not expressly invoked. More definitively, Bohr’s concept of complementarity, as developed after the Como lecture, does not allow for wave-particle complementarity as the latter has been commonly understood, by assuming that quantum objects can behave independently in the two mutually exclusive ways, either as waves or as particles. An attribution of any properties that would allow one to assume so would be precluded in strong RWR interpretations, such as Bohr’s ultimate interpretation, because these interpretations, in principle, preclude any conception of quantum objects or the ultimate reality responsible for quantum phenomena.

Bohr was always aware of the difficulties of applying the concept of physical waves to quantum objects or assuming that both types of behavior, particle-like and wave-like, pertain to the same individual entities, such as each photon or electron. While these difficulties might be handled in terms of wave-particle complementarity, they would virtually preclude applying to quantum objects the concept of wave-particle duality, which was widespread in the “old” (pre-quantum-mechanical) quantum theory, following de Broglie’s introduction of it in 1923. It is still advocated as possible or at least desirable in QM. (It is, again, inherent in Bohmian mechanics.) Following Born’s probabilistic interpretation of the wave function, Bohr spoke of quantum waves as “symbolic” in the following sense. Any concept formally or mathematically analogous to the representation of wave motion in classical physics only serves in QM as a mathematical symbol or set of symbols for the purposes of predicting, via Born’s rule, the probabilities of the outcomes of quantum experiments. As Bohr says in his reply to EPR, in considering the double-slit experiments,

Even if the momentum of [the] particle [considered] is completely known before it impinges of the diaphragm, the diffraction of the slit of the plane wave given *the symbolic representation of its state* will imply an uncertainty in the momentum of the particle after it has passed the diaphragm, which is the greater the narrower the slit.

[25] (p. 697).

Thus, this plane wave is not anything physical, but a formal concept that refers to the way of estimating the probabilities of quantum events observed in measuring instruments. In fact, this view begins to emerge even in the Como lecture, where one could still find a certain ambivalence in this regard and a shadow, a ghost, of the idea of wave or particle as something physical. Bohr speaks there of “the symbolic character of Schrödinger’s method,” in this respect no different from matrix mechanics [13] (v. 1. p. 66). This becomes Bohr’s persistent way of referring to the formalism of QM and its symbols, including ones formally analogous to those representing classical concepts. As he said, in the passage cited above, in justifying his concept of phenomenon,

Moreover, speaking in such a way [in terms of his concept of phenomenon] is just suited to emphasize that the appropriate physical interpretation of *the symbolic quantum-mechanical formalism* amounts only to predictions, of determinate or statistical character, pertaining to individual phenomena appearing under conditions defined by classical physical concepts [describing the observable parts of measuring instruments].

[13] (v. 2, p. 64; emphasis added)

Bohr’s ultimate, RWR, solution to the dilemma of whether quantum objects are particles or waves was that they were neither, any more than anything else, by the Heisenberg postulate. The postulate precludes de Broglie’s and related concepts of wave-particle duality (vs. Bohmian mechanics, where this duality applies ontologically to the ultimate reality considered, while Bohr’s complementarity applies at the level of observation.) Instead, either “picture” refers to one of the two mutually exclusive sets of discrete individual effects, described classically, of the interactions between quantum objects and measuring instruments, particle-*like*, which may be individual or collective, or wave-*like*, which are always collective, composed of discrete individual effects. The double-slit experiment is an example of the latter, as interference effects are composed of discrete traces of the collisions between the quantum objects considered and the screen in S2. The two sets of effects, observed in S1 and S2, may be seen as complementary in Bohr’s sense because they are mutually exclusive and require mutually exclusive experimental setups, on defining either of which one can decide.

As noted, the concept of wave-particle complementarity, or even duality, persists not only in interpretations or discussions of QM, but also in interpretations and discussions of Bohr’s concept of complementarity or his interpretation in general. Heisenberg often used wave-particle complementarity and sometimes argued it to be necessary (e.g., [37,42] and for criticism [16] (pp. 179–182]). The case is, however, subtle given that what Heisenberg calls the Copenhagen interpretation in the book is a complex mixture of his own and Bohr’s views, still in Heisenberg’s interpretation. It is also possible to see his concept of quantum waves, along the lines just indicated as, in the language in Heisenberg’s mentor Born, “probability waves,” reflecting the probability distributions defined by a given wave function, or in the language, just discussed, in his other, most important, mentor, Bohr, “symbolic waves.” More indicatively, in the comprehensive and up-to-date recent collection cited above [9], the only article questioning the role of wave-particle complementarity in Bohr is by the present author [43]. Most other articles make extensive use of wave-particle complementarity and sometimes duality. This is not to say that these articles do not offer important insights into Bohr or the physics and philosophy of quantum theory by exploring these concepts, let alone in general. I might cite, as sentimental favorites, [44,45], for their philosophical acumen and implications, but all articles there advance our understanding of Bohr’s thinking. Wave-particle duality and complementarity have also served important quantitative analyses of the uncertainty relations and complementarity, or the double-slit experiment, beginning with influential W. Wooters and W. H. Zurek’s article [46].

I stand by my argument concerning wave-particle complementarity as unnecessary and potentially misleading in quantum physics. As discussed below, Wooters and Zurek’s and subsequent quantitative findings could be reformulated without any appeal to either waves or particles as physical concepts (as they appear to be used by them, at least at the time), for example, by using them, following Bohr, as symbolic concepts or avoiding them entirely. On the other hand, while I am strongly inclined to see Bohr’s argumentation as avoiding, even if not in principle precluding, wave-particle complementarity, I shall refrain from arguing that alternative views are impossible. The present article is not the place to debate Bohr’s views and their development, which I have considered on several earlier occasions (e.g., [8,16] (pp. 179–352) [34] (pp. 107–172)). My argument is indebted to and, in many respects, follows that of Bohr (in my interpretation), but not in all respects, beginning with adding the Dirac postulate. More significantly, Bohr never considered the view of the double-slit experiment proposed here. Perhaps this was because he never considered the concept of entanglement, even in dealing with situations, such as that of the EPR experiment, defined by entanglement.

### 2.3. The Heisenberg-Von-Neumann Cut and Quantum Entanglement

As noted, Bohr saw the necessity of “discriminating in each experimental arrangement between those parts of the physical system considered which are to be treated as measuring instruments and those which constitute the objects under investigation … [as] a *principal distinction between classical and quantum-mechanical description of physical phenomena*” [25] (p. 701). By virtue of the cut, thus implied, this argument suggests that an object under investigation may be a classical object, defined here as an experimentally quantum object. It is, again, intriguing that Bohr himself never brought this argument to bear on the double-slit experiment, central to his argumentation concerning quantum physics, including in his reply to EPR, here cited. His counterargument to EPR was grounded in an extensive analysis of the double-slit experiment, which may be surprising as well, given that EPR’s argument does not appear to be connected to the double-slit experiment, never mentioned by EPR. For those familiar with Bohr’s work, this is not unexpected. For one thing, as, in Feynman’s words (Bohr effectively agreed), “the only mystery” of quantum physics, the double-slit experiment should underly all other quantum mysteries, including the mystery of the EPR experiment—the mystery of distant correlations without an action at a distance. Countering EPR, Bohr did not see the EPR experiment as implying the incompleteness of QM or else the possibility of an instantaneous action at a distance, “a spooky action at a distance,” as Einstein famously called it [47] (p. 155). Instead, Bohr saw the experiment only as “suited to emphasize how far, in quantum theory, we are beyond the reach of pictorial visualization” and ultimately beyond conception [13] (v. 2, p. 59). Bohr not only argued that the EPR experiment could be translated into a form of the double-slit experiment but also used the double-slit experiment to counterargue *EPR’s argument* concerning *the EPR experiment*. A proper analysis of Bohr’s argument is beyond my scope here. It was considered in detail from the RWR perspective in [7] (pp. 227–272). Essentially, Bohr argued that, if EPR’s claim to the effect that both the position and the moment of a quantum object can be assumed to be simultaneously *real*, even if they cannot be simultaneously measured (which they cannot by the uncertainty relations, as EPR acknowledged) is correct, it would lead to the disappearance of the interference pattern in S2 of the double-slit experiment. Its existence in S2 is, however, an experimentally confirmed fact, which implies that EPR’s argument cannot be correct.

One of the reasons that Bohr did not consider the cut in his discussion of the double-slit experiment in his reply may have been that his argument concerning the cut (he did not use the term), which occurs in closing, might have been added at a later stage of writing his reply. It appears to have been suggested by Heisenberg’s own unfinished reply to EPR, which was grounded in the question of the cut, indeed commonly referred to as the Heisenberg or the Heisenberg-von-Neumann cut [48]. Heisenberg decided not to proceed, apparently because Bohr’s reply was well underway. Bohr saw Heisenberg’s draft, and they corresponded on the subject, which might have suggested Bohr’s argument. In any event, as defining “a *principal distinction between classical and quantum-mechanical description of physical phenomena*,” this argument was crucial. According to Bohr,

This necessity of discriminating in each experimental arrangement between those parts of the physical system considered which are to be treated as measuring instruments and those which constitute the objects under investigation may indeed be said to form a *principal distinction between classical and quantum-mechanical description of physical phenomena*. It is true that the place within each measuring procedure where this discrimination is made is in both cases largely a matter of convenience. While, however, in classical physics the distinction between object and measuring agencies does not entail any difference in the character of the description of the phenomena concerned, its fundamental importance in quantum theory … has its root *in the indispensable use of classical concepts in the interpretation of all proper measurements*, even though the classical theories do not suffice in accounting for the new types of regularities with which we are concerned in atomic physics. In accordance with this situation there can be no question of any unambiguous interpretation of the symbols of quantum mechanics other than that embodied in the well-known rules which allow us to predict the results to be obtained by a given experimental arrangement described in a totally classical way.

[25] (p. 701; second emphasis added)

It is important to avoid two common misunderstandings of this and related statements by Bohr. The first arises by disregarding that, by adopting the Bohr postulate, Bohr refers only to the necessity of the classical description of the *observable* parts of measuring instruments, while assuming that they also have quantum parts through which they interact with quantum objects, thus enabling the emergence of quantum phenomena. This stratum and, as quantum, this interaction cannot be observed and, in RWR interpretations, are beyond representation or conception. This interaction is, in Bohr’s language, “irreversibly amplified” to the classical level of observable effects, such as a spot left on a silver screen [13] (v. 2, p. 73). The physical nature of this “amplification” is part of the problem of the *physical* emergence of classical reality from quantum reality or, in present terms, the reality ultimately responsible for quantum phenomena. In most assessments, including that of this author, this problem remains unsolved. There are arguments to the contrary, for example, on lines of decoherence or consistent histories (e.g., ref. [49] for an overview and references). These arguments have, however, been challenged and are not generally accepted. Fortunately, quantum phenomena and QM allow us to bypass this problem. As Bohr noted in 1937, QM is “justified only by the possibility of disregarding in its domain of application the atomic structure of measuring instruments themselves in the interpretation of the results of experiments” [12] (p. 88). This disregard, he added, may lead to new complexities in high-energy regimes [12] (p. 88). “The atomic structure of measuring instruments” is still disregarded in high-energy regimes, which may be responsible for the appearance of infinities and the necessity of renormalization in QFT. These complexities have not always been seen as adequately resolved, notwithstanding the great predictive capacity of QFT. QED is the best confirmed physical theory ever.

The second misunderstanding of Bohr’s view expressed in the statement under discussion is assuming that in Bohr’s interpretation, while observable parts of measuring instruments are described by classical physics, the independent behavior of quantum objects is *represented* by the formalism of QM. Bohr held no such view, at least not by the time of his reply to EPR in 1935. This view has been adopted by some, sometimes under the heading of “the Copenhagen interpretation,” as in Dirac’s and, especially, von Neumann’s classic studies, published in 1930 and 1932, respectively [36,50]. Both books, moreover, assume a classically causal independent behavior of quantum objects, with probability brought in by measurement. Bohr’s Como lecture of 1927, which introduced complementarity, may be seen as allowing and possibly adopting, ambivalently, this type of view and as having influenced both Dirac and von Neumann in this regard [7] (pp. 198–211). Bohr changed his views already under the impact of his first debate with Einstein in the Solvay conference, barely a month after the Como conference, which Einstein did not attend. In the passage under discussion, Bohr only says that classical theory cannot account for how quantum phenomena come about or predict the data observed. He does not say that QM represents the independent behavior of quantum objects or objects “under investigation,” which may be classical objects considered as experimentally quantum objects. As discussed above, the “symbols” of QM only have a probabilistically predictive role, without, by the Heisenberg postulate, offering a representation of how quantum phenomena come about, while these phenomena themselves are assumed to be represented by classical physics, by the Bohr postulate.

The circumstance that “the place within each measuring procedure where this discrimination is made is … largely a matter of convenience” is often seen as the *arbitrariness* of the cut. Bohr qualifies this claim, and this qualification is important, including, as will be seen, in the double-slit experiment. While “it is true that the place within each measuring procedure where this discrimination is made is … largely a matter of convenience,” it is true only largely, but not completely. This is because “in each experimental arrangement and measuring procedure we have only a free choice of this place within a region where the quantum-mechanical description of the process concerned is effectively equivalent with the classical description” [25] (p. 701). Accordingly, “the object under investigation,” an experimentally quantum object, is defined by how one decides to set an experiment, unlike classical physics or relativity, where this decision neither defines the object of investigation, always separated from the measuring instrument used, nor affects the outcome, correlatively to the absence of the uncertainty relations or complementarity. The quantum nature of this experiment is still defined by the ultimate, RWR, reality, the existence of which is manifested in or, in Bohr’s language, “symbolized” by Planck’s *h*, which, as a classically measured quantity, is not part of this reality [13] (v. 1, p. 53).

As in RWR interpretations, part of this reality, ontologically quantum objects and ontologically quantum parts of the instruments interacting with quantum objects are never on the observation side of the cut. Hence, they cannot serve as observational instruments. All observable properties are those of the observable parts of the instruments and, along with these parts, are described classically, by the Bohr postulate, while only appearing, as such properties, under the impact of quantum objects. The cut reflects the possibility of placing some classical parts of the overall arrangement in a quantum experiment, on either side of the cut, as “an object under investigation,” or in present terms, an experimentally quantum object concerning which predictions can be made by QM, as in the case the cat in the Schrödinger cat experiment or “the friend” in the Wigner’s friend experiment [4,5]. The case of D2 in the double-slit experiment in S2 is somewhat different. We do not make any quantum prediction concerning it based on its interaction with an object, O, traversing it. S2 and O form an entangled system, which, as explained below, cannot be separated into D2 and O at the time of this interaction. This entanglement and the rest of S2, of which D2 is part, only allows us to make predictions concerning O hitting the screen, which is a classical phenomenon, as is D2 considered by itself. These predictions are based on the initial position measurement of O, before it encounters D2, and the positions of both slits in D2. We predict the classical state of D2 after this interaction because this state is always the same and is known to us, and not on the basis of this entangled interaction as an observation, which this interaction is not. An observation is also an entanglement between the quantum objects considered and the instrument used, but of a different kind, because it leaves a trace in some observable part of this instrument, the trace that defines the data to be measured.

For an experiment to be quantum, the arrangement must include an ontologically quantum object, such as that traversing a slit in the double-slit experiment, a particle in radioactive decay and the decaying atom in the cat experiment, or the quantum object in the initial experiment set up by “Wigner” and “the friend,” on which the friend performs a subsequent measurement. An ontological quantum object is, again, unobservable as such, regardless of interpretation, and is beyond conception in strong RWR interpretations. On the other hand, D2, the cat, or “Wigner’s friend”, is always available to our thinking concerning them, even when, while hidden as is the cat or “Wigner’s friend,” not to our immediate sense perception. They can change their state: the cat can move around the box or may be dead, or the friend may know the outcome of the second experiment performed in the setup of the Wigner’s friend experiment. Nevertheless, while so changing, they can still be considered as the same and strictly distinguished from each other. One always deals with an idealization that is conceivable or, in some cases, observable independently of measuring instruments, vs. ontologically quantum objects. For one thing, as noted, while the measurement associated with variables such as location, momentum, or energy can be different, some ontologically quantum objects, such as elementary particles, are indistinguishable from each other in terms of their invariant characteristics, such as mass, charge, or spin. (Spin is purely quantum and hence only pertains to ontologically quantum objects.) Classical objects are always distinguishable from each other and are observable independently because the influence of measuring instruments can be neglected. In RWR interpretations, even invariant quantities associated with quantum objects are only observable as effects of the interactions between quantum objects and observational instruments. In these interpretations, ontologically quantum objects are always beyond thought, even at the time of observations (the only time when one can speak of them, if the Dirac postulate is assumed) vs. the traces left by their interactions with observational instruments. While to be considered in any way, ontologically quantum objects must be treated as experimentally quantum objects, they belong to the ultimate reality responsible for quantum phenomena. As such, they can never be on the observation side of the event or the cut. Hence, a quantum object can never be an instrument either, because, while having a quantum part interacting with ontologically quantum objects, an instrument must have an observable classical part for quantum effects to be observed. On the other hand, while it can be an experimentally quantum object when coupled to an ontologically quantum object, if considered by itself, a classical object, such as D2, the cat, or “Wigner’s friend,” cannot be treated by quantum physics. It can only be treated by classical physics if one assumes the Bohr postulate [4,5].

Importantly, in the present view, this is not a matter of the emergence of these objects as classical objects out of quantum objects at one or another point of the corresponding experiments. When considered by themselves, they are always (ontologically) classical objects; they can only be considered as experimentally quantum objects when coupled to ontologically quantum objects. Physically, classical objects do, of course, emerge from the microscopically quantum constitution of nature, such as elementary particles or quantum fields. What makes them classical is nature, in our interactions with it by means of our bodies and brains, which were made by nature to “see” the world as classical and not to “see” or even imagine anything ontologically quantum, which is nevertheless responsible for what we as classical, including in observational instruments in quantum experiments.

In certain circumstances, an (ontologically) quantum object considered by itself could be *treated* as a classical object, *without ever being* a classical object. Thus, when an electron is far from the nucleus (for large quantum numbers), its behavior can be treated classically, which represents the content of Bohr’s correspondence principle. This is, however, only a practical idealization that disregards possible quantum effects of this behavior, which could sometimes be observed, even beyond the electron’s spin, which is strictly quantum and can be observed only as such. In addition, this observed “behavior” is not that of an ontologically quantum object considered, but that of something observed, say, in a cloud chamber as a trace of the interaction between this object and the media of the instrument. This situation was considered in Heisenberg’s paper introducing the uncertainty relations, as well as by Bohr on several occasions [13] (v. 1, p. 85) [51] (pp. 72–76). At bottom, one still deals with the combination of stationary states (the states of constant energy and no longer orbits in QM) and discontinuous quantum jumps. These states are just too close to each other for this combination to be detected, but one would register these states (electrons or the photons they emit still cannot be “seen”) by “zooming” on them if one had an instrument to do so. Any such instrument would, however, need to be able, by interacting with electrons or “emitted” photons, to register quantum effects. An “emission,” too, is a classical concept, which cannot represent how photons are “emitted,” in RWR interpretations. All we can see are traces of photons, or what we assume to be photons, traces manifested, *visible*, in spectra. Similarly, as an ontological quantum object, a macroscopic quantum object (still defined as such by its microscopic quantum constitution), such as a Josephson device, can only be detected as quantum by means of a suitable instrument. Otherwise, it will be observed as a classical object and as such, something available to our immediate perception. In sum, an ontological quantum object, if considered by itself, can be treated both by quantum physics in all cases and by classical physics in some cases, without even being a classical object. A classical object, if considered by itself, can only be treated by classical physics, and can only be treated, as an experimentally quantum object, by quantum physics if coupled to an ontologically quantum object.

This article does not claim to offer a definitive treatment of the concept of quantum objects, which has been widely debated and remains unsettled. This article’s contributions in this respect are, first, an RWR form of this concept, accompanied by the Dirac postulate, and secondly, the possibility of considering, as in the case of O and D2, an entanglement between ontologically quantum objects and ontologically classical objects, treated as experimentally quantum objects. This entanglement is not between ontologically quantum objects and measuring instruments. The latter type of entanglement has been known beginning with Schrödinger’s introduction of the concept of entanglement, indeed, first as this type of entanglement, technically, an entanglement between quantum objects and quantum parts of observational instruments, while their observable parts are ontologically classical objects [6] (pp. 161–163). (O does not need to physically interact with D2 in either S1 or S2, although O may do so if it physically “touches” and “bounces” off a slit.)

The concept of entanglement, Schrödinger’s great discovery, is unique to quantum physics. It also exemplifies the view, inherent in the present interpretation of QM, that, while quantum phenomena are different from classical ones physically, the difference between QM and classical physics as theories is ultimately defined by abstract mathematical structures, including entanglement [52]. In establishing the difference between classical physics (both mechanics and electrodynamics) and relativity, the physical difference in the phenomena considered by these theories and the mathematical difference between these theories themselves go hand in hand. By contrast, in the present interpretation, the only physical connection between the formal mathematical structure of QM and quantum phenomena is that QM can predict the probabilities of the data observed in these phenomena, without representing either these phenomena or how they come about. Quantum phenomena are represented by classical physics (with the addition of special relativity in high-energy regimes) by the Bohr postulate, and how they come about is beyond representation or even conception by the Heisenberg postulate. Accordingly, the difference in the mathematics of classical and quantum theory is not connected to the physical difference between classical and quantum phenomena. Schrödinger was not happy with any of these features, including the Bohr postulate, which he debated with Bohr, as discussed in [4] (pp. 26–34), or, with QM itself, especially as interpreted along the RWR lines. He saw QM as “a doctrine born of distress” and “perhaps after all only a convenient calculational trick,” rather than a proper fundamental theory. He also agreed with EPR’s argument that, in view of the EPR experiment, QM was, while correct, incomplete, assuming locality (no action at a distance) [6] (pp. 154, 167). Schrödinger defined entanglement as follows:

When two systems, of which we know the states by their respective representatives, enter into temporary physical interaction due to known forces between them, and when after a time of mutual influence the systems separate again, then they can no longer be described in the same way as before, viz. by endowing each of them with a representative of its own. I would not call that *one* but rather *the* characteristic trait of quantum mechanics, the one that enforces its entire departure from classical lines of thought. By the interaction of the two representatives (or ψ-functions) have become entangled. To disentangle them we must gather further information by experiment, although we knew as much as anybody could possibly know about all that happened. Another way of expressing the peculiar situation is: the possible knowledge of a *whole* does not necessarily include the best possible knowledge of all its *parts*, even though they may be entirely separate and therefore virtually capable of being ‘best possibly known,’ i.e., of possessing, each of them, a representative of its own. The lack of knowledge is by no means due to the interaction being insufficiently known—at least not in the way that it could possibly be known more completely—it is due to the interaction itself.

[53] (p. 555)

Formally, in considering two separate quantum systems, each defined by the corresponding wave function, ${|\left.\psi \right\rangle }_{1}$ and ${|\left.\psi \right\rangle }_{2}$ (representing pure states), providing, in Schrödinger’s terms, “maximal expectation catalog for each,” thus the maximal possible knowledge concerning the future of each system, one can also define [35], (p. 161), one can define the combined wave function, providing such a catalog for the combined system. This wave function is just the tensor product ${|\left.\psi \right\rangle }_{1}⊗{|\left.\psi \right\rangle }_{2}.$ “But,” Schrödinger discovers, “the converse is not true. Maximal knowledge of a total system does not necessarily include total knowledge of all its parts, not even when these are fully separated from each other and at the moment are not influencing each other at all” [35] (p. 161). It is not when one deals with an entanglement, in which case the combined state cannot be factorized in this way. Thus, if one considers two quantum objects, A and B, with possible pure states $|\left.\psi_{A1}\right\rangle$ and $|\left.\psi_{A2}\right\rangle$, and $|\left.\psi_{B1}\right\rangle$ and $|\left.\psi_{B2}\right\rangle$, the state $\left.{|\psi }_{A1}\right\rangle ⊗\left.\psi_{B1}\right\rangle +\left.{|\psi }_{A2}\right\rangle ⊗\left.\psi_{B2}\right\rangle$ is entangled and hence not factorizable into a tensor product of two states [28] (p. 23). If the combined system is in this state, one cannot define a pure state for either system as a separate system. Accordingly, as Schrödinger says, “the possible knowledge of a *whole* does not necessarily include the best possible knowledge of all its *parts*, even though they may be entirely separate and therefore virtually capable of being ‘best possibly known,’ i.e., of possessing, each of them, a representative of its own.”

A “representative” is a ψ-function associated with a quantum object. This need not mean, and in RWR interpretations, does not mean that it *represents* this object in a realist sense, but only that a ψ-function can be used for predicting the probabilities of experiments on this object. Schrödinger’s point that “the lack of knowledge is by no means due to the interaction being insufficiently known—at least not in the way that it could possibly be known more completely—it is due to the interaction itself” is amplified by strong RWR interpretations so that the expression “the lack of knowledge” is no longer applicable. This is because, in these interpretations, when it comes to the ultimate reality responsible for quantum phenomena, and hence, for the situations defined by entanglement, at stake is not merely the lack of knowledge concerning this reality but the impossibility of forming a conception of it. Quantum probability, including in cases of entanglement, is also defined by this impossibility rather than any lack of knowledge concerning this reality.

Quantum phenomena themselves are never entangled. They disentangle the entanglement considered by transforming the physical situation defined by this entanglement, after the corresponding preparation and the phenomena established by it (which are not entangled either), back to the observation of separate quantum phenomena. In contrast to complementarity or the uncertainty relations, entanglement is not a feature of quantum phenomena. It is a defining mathematical feature of QM which enables certain specific predictions, such as, and, in particular, quantum correlations. It is conceivable that these correlations or other experimental features predicted by using entanglement could be predicted by a QT that does not contain the mathematics of entanglement. However, if one uses QM or QFT, entanglement is a mathematical part of it, related to physics only in terms of probabilistic predictions of what is observed, classically, as quantum phenomena.

Speaking of entangled quantum objects requires caution as well. In RWR interpretations, one cannot rigorously speak of the *interaction* between quantum objects that occurred before an observation, because one cannot rigorously speak of what happens between experiments. In addition, in the present view, by the Dirac postulate, the concept of quantum objects only applies at the time of observation or measurement, technically, again, preceding the act of observation. Thus, the combined entangled system (as a whole) precludes a sufficient knowledge of each part to define each as a separate object, which is impossible in the present view and is one of the motivations for adopting the Dirac postulate. An entangled “whole” invoked by Schrödinger has no parts. In what sense is it a “whole” then, a whole of what? At the time of entanglement, there is only an (RWR) physical reality, ultimately responsible for quantum phenomena, but by creating a phenomenon or phenomena predicted by the mathematics of entanglement, a measurement or measurements will disentangle this reality. One deals with a particular state of the ultimate reality responsible for quantum phenomena that give rise to a particular type of these phenomena and effects, specifically correlations, such as those observed in the EPR-type experiment or in the interference pattern in the double-slit experiment.

This situation fully applies when one of the “entangled” entities is the quantum part of a measuring instrument that has “interacted” with the quantum object considered, as the other part of this entanglement. This interaction itself, unobservable as such, always precedes the observation, defined by, to return to Bohr’s language, the “amplification” of this quantum interaction to the classical level of observation, defining quantum phenomena. As noted, the concept of entanglement was initially introduced by Schrödinger to account for this entanglement and then extended by him to apply to independent quantum objects, specifically in the EPR-type experiment [6] (pp. 161–163).

This extension requires qualifications due to the role of observational instruments, especially in RWR interpretations, with Bohr and Dirac postulates, adding further complexities. Under these assumptions, one deals with two quantum objects, *S*_1_ and *S*_2_, associated with two preparatory measurements performed prior to a certain unobservable and unknowable or even inconceivable (RWR) reality defined by this preparation, and then two quantum objects, $S_{1}^{\prime }$ and $S_{2}^{\prime }$, associated with two measurements performed subsequently. The second set of measurements is predicted by the mathematics of entanglement in the basis of the first set (usually in terms of statistical correlations in repeated experiments with the same preparation). Technically, by the Dirac postulate, $S_{1}^{\prime }$ and $S_{2}^{\prime }$ are not the same as $S_{1}$ and $S_{2}$, but in QM, one can treat them as statistically the same. In fact, as noted, this does affect the situations, because one only deals with the outcomes of measurements, which will be the same whether one treats these (indistinguishable) objects as identical or different. Any quantum entanglement, as a mathematical procedure, can be defined strictly in accord with this situation. Knowing the outcome of $S_{1}^{\prime }$ in an EPR-type experiment, say that of the position, with value Q, allows one to predict exactly, with probability one, the outcome of the position measurement on $S_{2}^{\prime }$. This prediction remains conditional because, as explained, one can always perform a complementary measurement, that of the momentum, with value P, instead, which measurement annuls that prediction because there is no way of ever verifying it, once this alternative measurement is performed. A subsequent position measurement on $S_{2}^{\prime }$ will yield a value different from Q. This fact, which exemplifies complementarity, was underappreciated by EPR, as was the role of complementarity or measuring instruments in QM [7] (pp. 227–272).

Schrödinger’s view of this situation *as not merely*, *“one* but rather *the* characteristic trait of quantum mechanics, the one that enforces its entire departure from classical lines of thought,” may appear to be in conflict with Bohr’s view that “this necessity of discriminating in each experimental arrangement between those parts of the physical system considered which are to be treated as measuring instruments and those which constitute the objects under investigation may indeed be said to form a *principal distinction between classical and quantum-mechanical description of physical phenomena*.” There is, however, no conflict between these views. First, Bohr speaks of “*a* principal distinction,” which may allow for other such distinctions. Whether there is a single defining distinction between classical and quantum physics is under debate. In any event, these two distinctions are related or even correlative because the interaction between a quantum object and an observational instrument is a form of entanglement. This is the case even if, by the Bohr postulate, quantum instruments are described classically in their observable parts, because these instruments also contain quantum strata through which they interact with quantum objects. These quantum strata are responsible for the entanglement between observational instruments and quantum objects, which, physically, makes possible all quantum predictions by using QM (cum Born’s rule) [7] (pp. 192–196).

In the present view, then, an entanglement not only precludes defining any of its entangled quantum parts separately but also from speaking of such parts, and, if one assumes the Dirac postulate, from defining the entangled system as a quantum object in the first place. To do so would, by the Dirac postulate, require an observation, and no entanglement, while in place, can be observed. Instead, one deals with a particular form of the ultimate reality responsible for quantum phenomena, which leads to a particular type of effect, predicted by the mathematics of entanglement. This mathematics does have parts, state vectors, $|\left.\psi_{A1}\right\rangle$ and $|\left.\psi_{A2}\right\rangle$, and $|\left.\psi_{B1}\right\rangle$ and $|\left.\psi_{B2}\right\rangle$, associated with each object considered, by using which a linear combination of tensor products, like $\left.{|\psi }_{A1}\right\rangle ⊗\left.\psi_{B1}\right\rangle +\left.{|\psi }_{A2}\right\rangle ⊗\left.\psi_{B2}\right\rangle$, enables one to predict (based on the data of the pre-entanglement experiments) the probabilities of the possible states of two objects considered after the entanglement. The ultimate reality, however, which is responsible for the phenomena thus predicted or observed (thus disentangling this entanglement), has no parts comprising it. In fact, no concept of wholeness applies to this reality either. This reality is unobservable, regardless of interpretation, and in RWR interpretations, is beyond conception. This is in accord with Bohr’s view of the EPR experiment as “suited to emphasize how far, in quantum theory, we are beyond the reach of pictorial visualization,” rather than showing the incompleteness of QM or else its nonlocality, allowing for an action at a distance [13] (v. 2, p. 59). We may be confronting a reality that is beyond the reach of thought altogether.

## 3. Classical Observation and Quantum Interaction in the Double-Slit Experiment

**Figure 2:**
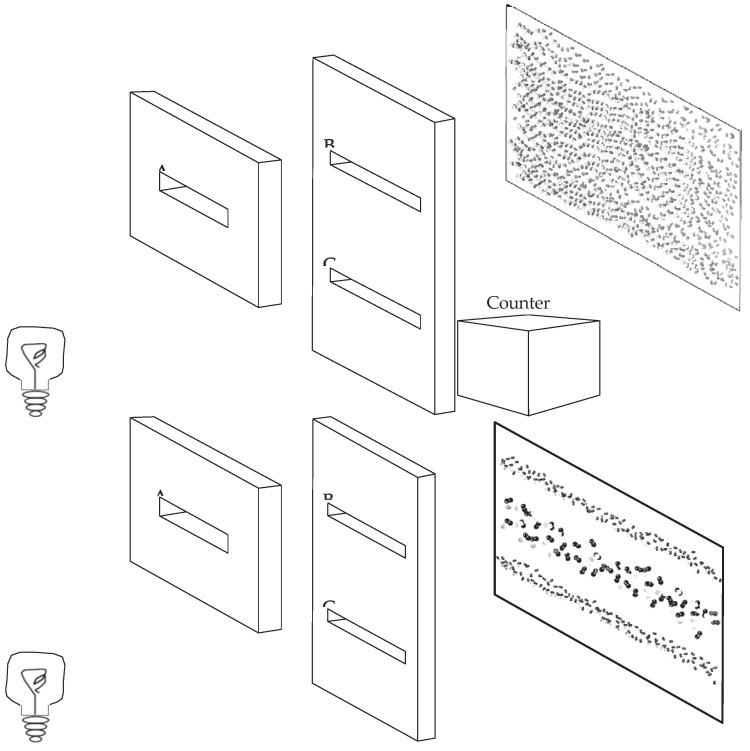
Two setups, S1 and S2, of the double-slit experiment are depicted as symbolically representing, following Bohr [12] (v. 2, pp. 48–49), that one can only see the (“heavy,” humanly made) measuring devices and the traces left by the interactions between them and quantum objects, but not what happens in between, which cannot be represented or even conceived of. The dots in first drawing, representing S1, are randomly distributed. The dots in the second drawing show the appearance of the interference paMern in S2, after many repeated runs of the experiment.

The preceding argumentation grounds this article’s rethinking of the double-slit experiment by considering two fundamentally different functionings of the diaphragm with slits, D2, in S1 and in S2: as a classical object, in fact part of the measuring arrangement in S1, and as an experimentally quantum object in entangled interaction with the ontologically quantum object, O, traversing the diaphragm, in each run of the experiment in S2. As explained in Section 2, considering D2 as an experimentally quantum object is possible by virtue of applying the Heisenberg-Von Neumann cut to D2 in S2, because O is an ontologically quantum object. The interference pattern can only appear if O is ontologically quantum, even if possibly composite and relatively large, such as a fullerene C60 molecule. Like any entanglement, this entanglement is unobservable at the time it takes place, and, in the strong RWR interpretation assumed here, is not only unrepresentable but also inconceivable. In S1, the interaction between D2 and O is an entanglement as well, but in this case as an event of quantum observation and measurement (which is, as explained in Section 2.3 always an entanglement), in which D2 functions as a measuring instrument with the position of the slit as the measurement of the position of O in traversing D2. Hence, D2 is on the measurement side of the cut, rather than on the object side, as it is in S2. The observable effects of the entanglement, in each run of the experiment, between O and S2 are statistical correlations, composed of random individual effects in each run itself. Two setups (S1 and S2) are shown in Figure 2. 

Technically, one does not need the diaphragm, D1, with a single slit. Following Bohr, however, the observation or measurement M1 of O’s interaction with the first diaphragm, rigidly fixed to the support, with a single slit as the “preparation,” PS1 or PS2 (which is M1 in S1 or S2), conveniently defines the position of O for each run of the experiment in either S1 or S2 [13] (v. 2, p. 48) [25] (pp. 697–698). O can be assumed to be an object with a known momentum, before its encounter with D1. Once it is established to be beyond D1 (rigidly fixed to the support), so that O’s position is that of the slit (assumed to be sufficiently narrow), during the interaction, its momentum is no longer known by the uncertainty relations. As explained in Section 2.1, an observation and a measurement are two separate procedures in dealing with quantum phenomena. In this case, the measurement is defined by the position of the slit, and for simplicity, I shall hereafter just refer to measurement, M. An observation or a measurement, then, always takes place after the interaction between the object and the instrument considered has taken place and is based on a trace of this interaction. In the case of M1 (or M2_S1_ in S1), this trace is assumed to coincide with the position of the slit, in this case known, as the positions of other slits, in advance. One can consider the location of any such position, say, that in D1, as a measurement only after one knows that O is beyond the slit, by the click of the counter at D2 in S1, and by the trace of the screen in S2. Neither of these events is guaranteed in a single run of the experiment, although the probability that either event will occur is high. The same applies in the case of O passing one or the other slit in D2 in S1, with the position of the slit and thus of O known in S1. The size of the slit represents the uncertainty in the coordinate Δ*q*, but the slits can be assumed to be sufficiently narrow for this uncertainty to be negligible. They need to be sufficiently narrow, while the distance between the slits needs to be sufficiently large, for the interference pattern to be detected in S2.

The registered events in the experiment are defined as measurements, although, in S1, M2, registering through which slit of D2 O has passed, becomes a new preparation, which redefines the final event M3, when O hits the screen, in each run. The “interaction” between the particle and the diaphragm (D1 or D2), usually described in classical (and hence in the present view, inapplicable) language as O’s “passing through the slit,” is unobservable, and in RWR interpretation is unrepresentable or inconceivable, and is always an entanglement, even in (technically preceding) M1 in S1 and S2, and M2 in S1. The exact time of this interaction is not known either, which is true in all such interactions. What can be determined exactly is the time of occurrence of the observation or registration of a quantum phenomenon, say, as defined by a movement of a pointer of the instrument, still, importantly, under the condition of the time-energy uncertainty relations. The latter will preclude one from exactly measuring the energy of the object. If, however, one knows, for example, by the trace on the screen, that the particle is on the other side of D1 or, in S1, D2, *which is only possible after this interaction*, this can be considered as a measurement (defined by the position of the slit). Technically, P must, and implicitly does, include the whole experimental arrangement, different (PS1 or PS2), depending on whether P is part of S1 or S2. In accordance with the structure of quantum observation and measurement, as considered in Section 2.1, one deals with two phenomena corresponding to two events considered, preparation, P, and measurement M, within the overall experimental arrangement, S1 or S2, of the experiment. In S1, there are three such observed phenomena or events, M1 (PS1), associated with D1; M2, associated with D2, registering the slit of the interaction between D2 and the quantum object considered; and M3, which is an observation–measurement of the trace on the screen. In S2, where there are only two registered events, M1 (PS2), associated with D1, and M2, an observation–measurement of the trace on the screen.

Following M1 (P) (which is the same in S1 and S2), in S1, the interaction between O and D2 is a classically described event of observation and then measurement, M2_S1_, with the diaphragm, D2, considered as part of the measuring apparatus and observed as a strictly classical object. This event is followed by another event of observation and then measurement M3_S1_, that of registering the trace left by O on the photographic screen. One does not know when O passed the slit, and it is possible for it to traverse the slit more than once before it is registered by the detector. The latter and several other quantum aspects of the situation can, however, be disregarded as statistically negligible. M2_S1_ can, again, be considered as a new preparation, with M3_S1_ as an observation. Thus, each individual run of the experiment in S1 contains the sequence of three events:

M1_S1_ → M2_S1_ → M3_S1_

By contrast, in each individual run in S2, the “passing” of O beyond D2 cannot be associated with an observable event and thus with any measurement. If it could be, the interference pattern would disappear. Only O’s interaction with the screen, leaving a trace, a spot, on the screen, can be associated with a measurement. Passing through the diaphragm is an unobservable entangled interaction between two quantum objects, O, as an ontologically quantum object, and D2, as an experimentally quantum object. As explained, considering the diaphragm as an experimentally quantum object is possible by using the Heisenberg-von Neumann cut, given that D2 is coupled to an ontologically quantum object, O, a coupling that is required for an ontologically classical object to be considered as an experimentally quantum object. This interaction cannot be observed and hence registered. No quantum interaction, entangled or not, can be. Only its effects can. What we *see*, in any sense one can associate with this word (observe with our eyes, hear as clicks, imagine as visible in our mind’s eye, and so forth) is only D2 or the whole arrangement of the experiments, as a classical object, without any changes in the situation after O is, presumably, beyond the diaphragm on its way to the screen. The only observed change after M1_S2_ is M3_S2_, manifested as a new spot on the screen. In S1, D2 is the same, but counters or other means of registering O in the immediate vicinity of D2 make this an event of observation or measurement. In S2, there is no such change in the immediate vicinity of D2, and hence no observation, but only a quantum interaction. We cannot *see* this interaction, but it affects the outcome of the experiment because the positions of the slits in D2 define the interference pattern observed in multiple runs of the experiment. The only registered event of observation or measurement, after the preparation, M1_S2_ in S2, is M2_S2_, defined as the trace left by O on the screen, similar to M3_S1_ in S1. Indeed, one cannot know which arrangement was used, S1 or S2, on the basis of a single trace or a small number of traces (before the interference pattern appears). The sequence of observed events in S2 is

M1_S2_ → M2_S2_ (vs. M1_S1_ → M2_S1_ → M3_S1_ in S1)

At the final stage of S2 after M2_S2_, the apparatus, in its observable parts, including D2, is treated as a classical system. By contrast, at the time when O, as an ontological quantum object, traverses D2 and in this sense is in an interaction, an entanglement, with D2, D2 is treated as an experimental quantum object. The slits can be wide enough for O to pass through them without touching D2 in some individual runs, without changing the interference pattern of traces in multiple runs in S2, or, for that matter, the random pattern in S1, where such passings can in fact be detected. They cannot be in S2. I reiterate that, in the present view, one is not dealing with D2 as reemerging as an ontologically classical object from an ontological quantum object after this interaction or emerging as an ontologically quantum object from a classical object during this interaction. D2, when considered by itself, is always an ontologically classical object, but it can be treated as an experimentally quantum object during the time of its interaction with O, as an ontologically quantum object. This time interval cannot, again, be defined exactly. All that can be known is that this interaction happened after O passes D1 and before it hits the screen.

**Figure 3:**
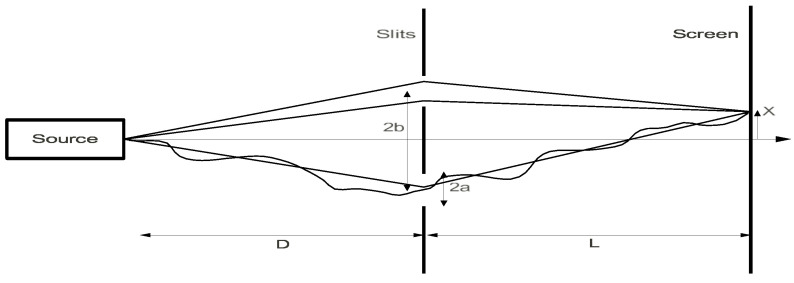
The diagram of probability calculation in S2, following [54] (p. 1027, Figure 2). In the present setup of the experiment, the source is replaced with the diaphragm D1 with one slit.

To calculate the probability for S2 correctly, one must (speaking in classical terms) assume that O can reach the screen, corresponding to M2_S2_, not merely by passing either one slit or the other, but also by passing both, an event that has never been registered. For one thing, if the distance between slits is sufficiently large, while still allowing for the interference pattern to emerge, and the distance between D1 and D2 is sufficiently small, this “spreading” of O between slits could violate relativity. In any case, no such event can be assumed on experimental grounds, as things stand now. More formally, in S2 the probability of O reaching the point X on the screen will be, considering for simplicity the one-dimensional case, following [54] (p. 1027), as showing in Figure 3:

P (x; a, b) = P_1_ (x; a, b) + P_2_ (x; a, b) + I_12_ (x; a, b),

where P_1_ (x; a, b) and P_2_ (x; a, b) are the probabilities for O passing through, respectively, the first and the second slit, and I_12_ (x; a, b) is the probability interference term. 2a is the width of the slits and 2b is the distance between slits. In terms of probability amplitudes,

I_12_ (x; a, b) = A_1_(x; a, b)A_2_(x; a, b) * + A_2_(x; a, b)A1(x; a, b) *

I refer to M. Beau’s calculations because they use all the quantities that need to be considered for making actual predictions in this case. These calculations, while relatively elementary, are not automatic and are quite elaborate, and they are helped by Feynman’s path-integral formalism [54] (p. 1035). On the other hand, the above diagram in Beau (Figure 3) could be, and in the present view is, misleading, by suggesting particles’ “paths” and wave and particle motions. Following Bohr’s drawings, my diagram above (Figure 2) would only contain X, the point of a trace on the screen, predicted by means of the formalism of QM. Feynman’s path integral approach does not imply either actual physical paths or wave or particle motions, beyond using them as a heuristic visual help (just as are Feynman diagrams) in estimating the probabilities of the outcomes of quantum experiments. The overall probability amplitude for a given quantum event is calculated by adding, *integrating*, all possible contributions in all ways in “paths” in *the configuration space* in which events can be probabilistically estimated, but not anything like trajectories of particles or wave motions in physical space and time. If one tries to associate, on classical lines, these configuration-space trajectories with those in space and time, some of such trajectories will be not only bizarre but strictly impossible. The contribution of each such path is proportional to $e^{\frac{iS}{\hbar }}$, where *S* is the action, which is the time integral of the Lagrangian along the path, again, in the configuration space. No description of anything between registered events is necessary or assumed in RWR interpretations, and in fact is in principle precluded in them. Feynman’s (Lagrangian) formalism is equivalent to the standard Hamiltonian, say, Schrödinger’s formalism, but it offers more effective ways of calculations in some cases, even in QM. It is especially helpful in QFT, to which Feynman made his main contributions, which, as noted, brought him his Nobel prize for the renormalization of QED (shared with J. Schwinger and S-I. Tomonaga). One can also dispense with the wave-particle conceptual grounding of calculations in Wooters and Zurek’s influential article [46] and other works and commentaries.

As I argue here, however, it may not be possible to avoid considering the quantum interaction between O and D2, as an experimentally quantum object in explaining the double-slit experiment. The presence of the interference term above

I_12_ (x; a, b) = A_1_(x; a, b)A_2_(x; a, b) * + A_2_(x; a, b)A1(x; a, b) *

in describing the state in the sense of the formalism, say, the wave function associated to O, ${|\left.\psi \right\rangle }_{O},$ after it passes D1, indicates that the combined state of the interactive system ${|\left.\psi \right\rangle }_{D2+O}$ is not factorizable: ${|\left.\psi \right\rangle }_{D2+O}$ $\neq {|\left.\psi \right\rangle }_{A}⊗$ ${|\left.\psi \right\rangle }_{B}$, associated with O and D2, respectively. This state is entangled. Because the physical state of D2 remains the same, it can be considered as predicted with probability P_D2_ = 1 after the interaction with O, say, by the trivial pure-state wave function,${|\left.\psi \right\rangle }_{D2}$. Qualitatively, there is an interaction between O (an ontologically quantum object) and D2 (an experimentally quantum object), because the probabilities of M3_S2_ and defined by it, and the fact that one cannot define each object at the time of this interaction or the wave function of each separately. These are the defining features of quantum entanglement. Accordingly, it would be difficult to see this interaction otherwise. Admittedly, this is not a rigorous formulation of ${|\left.\psi \right\rangle }_{D2+O}$, which would be quite difficult to construct, as it usually is when dealing with continuous variables. The original EPR state, which is a subtle construction, cannot be performed in a laboratory because it is not normalizable. While Schrödinger discusses entanglement for the interaction between the instrument and the quantum object considered in detail, he does not construct any such entanglement formally and uses the EPR experiment as a formally defined example of entanglement. One must keep in mind that D2 in S2 is not a measuring instrument! Most entangled states have thus far been constructed in quantum information theory, uniformly for discrete variables and finite-dimensional Hilbert spaces, which are discussed in textbooks on quantum information theory in dealing with quantum entanglement. While quantum-informational methods are general, using them in dealing with continuous variables, including in considering entanglement, remains difficult, not the least mathematically, because they require infinite-dimensional Hilbert spaces. It is, accordingly, not surprising either that quantum information theory has been rarely, if ever, used in considering the double-slit experiment, as opposed to the EPR experiment in Bohm’s version for discrete variables, and related subjects such as quantum correlations, Bell’s theorem, the Kochen–Specker theorem, and related findings. The contribution of quantum information theory to understanding these problematics, including the role of entanglement in them, has been significant and sometimes ground-breaking, including in derivations of QT (for discrete variables) from first principles, as to give two arguably most significant examples [55,56]. One of the reasons for the difference between discrete and continuous variables in this regard may be that in the case of discrete variables, which are purely quantum, one only deals with ontologically quantum objects. In the case of continuous variables, experimentally quantum objects that are ontologically classical need to be more involved, as they are in the double-slit experiment. Continuous variables make entanglement “messy,” physically and mathematically; discrete variables make it clean. Having discussed quantum information theory on many occasions (including in several books and articles cited here), I am aware of the difficulties of dealing with continuous variables in this field. On the other hand, as this article exemplifies as well, there is no QT, certainly QM, let alone QFT, without continuous variables. We must, accordingly, understand them in dealing with quantum entanglement, introduced for continuous variables in the first place. The double-slit experiment, I argue, offers us new ways of doing so.

In sum, it is the role of D2 as an experimentally quantum object in its quantum interaction, as an entanglement, with O, as an ontologically quantum object, in S2, that defines the double-slit experiment as a quantum experiment, still, to return to Mermin’s phrase, “the greatest of all quantum conundrums” [10] (p. 108). The interaction between O and D1, in both S1 and S2, or D2 in S1, is still quantum and from an entanglement, but in this case, D1 or D2 in S1 function as part of the measuring instrument registering an event, which is not the case for D2 in S2. S1 is also quantum insofar as the outcome of each run of the experiment, after registering M2, is defined determinately by the interaction between O and a given slit, can only be predicted by QM (or possibly some other QT), and not by classical physics. The difference with S2 remains essential, however, because, vis-à-vis S1, there is no measurement of O at D2, and yet the presence of D2 affects the outcomes. If, instead of O, as an ontologically quantum object, we had a small classical object that would not have physically interacted (“touched”) the diaphragm, its future behavior would not be affected: nothing changes. If it had physically interacted with the diaphragm, it would just be a new measurement, allowing one to predict, ideally deterministically, where it would hit the screen. In dealing with the double-slit experiment as a quantum experiment, in which case O is ontologically quantum, any prediction concerning where it would hit the screen could always be probabilistic in S1, just as it will be in S2. These probabilities will, however, be calculated differently in S1 and S2 because there is no measurement (like M2 in S1) in the vicinity of the diaphragm in S2, in accordance with the following fact, noted by Bohr, reflecting the correlational nature of the interference pattern. He said:

The probability of the electron reaching a given element of area on [the photographic] plate is determined not by the presence of any particular slit, but by the positions of all the slits of the … diaphragm [This] fact would be quite incompatible with drawing … conclusions regarding the “course” of such phenomena—say through what particular slit of the second diaphragm the particle passes on its way to the photographic plate.

[25] (pp. 697–698).

A mere presence of D2 changes what we see on the screen in S2 due to the impossibility of any knowledge of how O gets over D2, with which, as an experimentally quantum object, O, as an ontologically quantum object, interacts in a quantum entanglement, on its way to the screen. A path-integral calculation would reflect this difference as well.

As stated from the outset, it is of some interest that even this difference, let alone the fact that one deals in this case with the *quantum* interaction and entanglement between O and S2, were not previously considered in the massive literature on the double-slit experiment, at least to my best knowledge, including extensive internet search while working on this article. Bohr, again, might have been aware of this difference, or at least the reason for it, but he never commented on it or on the quantum interaction between O and D2 in considering the double-slit experiment. In his reply to EPR, he did note that some parts of the apparatus in the double-slit experiment could be treated as “objects of investigation” (to which the uncertainty relations must apply) rather than parts of the measuring apparatus [25] (pp. 697–698). But he only applied this insight to the case of measurement, without considering D2 as a quantum object in the absence of an observation or measurement in S2. This might have been because his primary concern was the impossibility of considering, as EPR did, the reality of quantum variables independently of the measuring arrangements which, and, according to Bohr, only which, could establish this reality.

While dealing with *the situations of entanglement*, as in the case of the EPR experiment, Bohr never considered *the concept of entanglement* either. The concept, introduced by Schrödinger, was extensively discussed by him in three articles written in response to EPR’s paper [6,53,57] and following Bohr’s reply, a draft of which Schrödinger read while working on this paper, as discussed in [4] (pp. 26–34). It is not clear how familiar Bohr was with these articles, and if he was, he might have been skeptical about them because Schrödinger’s discussion of the EPR experiment agreed with EPR’s argument, challenged by Bohr’s reply. If so, while he was, in my view, right to be skeptical about this part of Schrödinger’s argument, Bohr still missed entanglement. This concept, which is consistent with Bohr’s argument in his reply to EPR, was a crucial new concept, essential in defining the difference between quantum and classical physics, as, in Schrödinger phrase “not *one* but the characteristic trait of quantum mechanics, the one that enforces its entire departure from classical lines of thought” [53] (p. 555). This is part of my argument as well, supported by the double-slit experiment and even defining it, consistently with the three postulates grounding the present interpretation—the Heisenberg, Bohr, and Dirac postulates. Entanglement is not a postulate: it is a feature of the formalism predicting quantum phenomena, as quantum correlations, similarly to the way the commutators in the formalism predict the uncertainty relations. Entanglement can be used to define a postulate, even, in accord with Schrödinger’s view, distinguishing quantum theory from classical theory, as was carried out, under the heading of “the purification postulate,” in quantum-informational terms, for QT (in finite dimensions) for discrete variables in [56], as discussed in [52] (pp. 24–26) Schrödinger, on the other hand, did not appear to have been interested in the double-slit experiment or commented on it. Thus, two founding figures most likely to have reflected on the aspect of the double-slit experiment considered here also had reasons to miss it.

Heisenberg’s discussion of the double-slit experiment in considering “the Copenhagen interpretation” (his version of it) does not expressly comment on this aspect either. This discussion, however, adds an helpful angle, by linking the experiment, and specifically, the role of D2 in S2 in arguing, in the passage cited earlier, that “there is no description of what *happens* to the system between the initial observation and the next measurement,” and that one “would get into hopeless difficulties if one tried to offer” such a description [37] (p. 52). Heisenberg shows that assuming that one can observe an interaction between O and D2 in S2 would imply a contradiction because the resulting probability distribution would be incompatible with observing the interference pattern, which one always observes in S2. “Any attempt to make such a description,” he said, “would lead to contradictions; this must mean that the term ‘happens’ is restricted to the observation” [37] (p. 52). This is consistent with the present, strong RWR view, which, as discussed, Heisenberg replaced with a mathematical realism at this stage of his thinking, thus allowing for what happens between experiments to be described by the formalism, such as the probability function. As he says, “this example [that of S2 of the double-slit experiment] shows clearly that the concept of the probability function does not allow a [physical] description of what happens between two observations” [37] (p. 52). Heisenberg then argued, as I do here, that, unlike in S1, the interaction between O and D2 in S2 cannot be treated as an observation. What Heisenberg did not argue and appears to have missed, and what I do argue here is that this interaction can be treated as an entanglement between an ontologically quantum object, O, and an experimentally quantum object, D2. Any observation, such as M3_S2_, on the screen, disentangles it while preserving the interference pattern. If one instead makes an observation allowing one to know which slit O traversed in each run of the experiment, the interference pattern will be destroyed.

Heisenberg, in his discussion of the double-slit experiment or quantum physics in general, in this book and elsewhere, also extensively relied on wave-particle complementarity [37] (pp. 45–46). By contrast, this article aims at removing the idea of wave-particle complementarity from quantum physics as unnecessary and even misleading, and possibly contrary at least to the spirit of Bohr’s concept of complementarity and his understanding of quantum physics, while admitting alternative views of Bohr’s thinking as possible. The double-slit experiment, I have argued here, justifies this removal. As I have also argued here and elsewhere, nature has no waves or particles, and hence neither wave-particle duality nor wave-particle complementarity. We do have these concepts, and there is no need to abandon them, any more than classical physics, where these concepts are so useful. While these concepts can also apply to quantum phenomena, described by classical physics, their application there is limited and requires a re-delimitation and even redefinition of either concept, for example, by replacing the wave-interference pattern with that of the correlations of discrete phenomena or events in the double slit-experiment. By contrast, the ultimate reality responsible for quantum phenomena may be, and in the RWR view, is beyond the reach of either concept. But then, in the RWR view, this reality is beyond the reach of all concepts, while, importantly, allowing one to apply these concepts at the level of such effects, which QM or QFT can predict, fully in accord with what is experimentally observed, as things stand now.

## 4. Conclusions

This article primarily addresses physical and philosophical questions, some of which do, however, involve mathematical foundations of QT, discussed in detail from the RWR perspective in [52]. As discussed there as well and as the present article confirms, especially by considering the fundamental role of human decision in defining quantum experiments, specifically in the case of complementarity, the RWR view also implies a change in the character of experimental physics. Experimental physics is no longer defined, as in classical or relativistic physics, in tracking the independent behavior of the systems considered and measuring their independent properties. It is defined by creating new arrangements of experimental technology reflecting the fact that what happens in quantum experiments is *unavoidably* defined by what kinds of experiments we decide to perform, by how our interactions with nature affect physical reality by a unique act of observation as creation. I qualify by “unavoidably” because, while the phenomena observed in classical physics or relativity may be affected by experimental technology and while we do stage experiments there, one can, in principle, observe these phenomena without affecting what is observed, which allows one to treat these phenomena as ideally representing the corresponding objects. Thus, one still follows what happens in any event. By contrast, in the present view, a quantum experiment is a creation of a new technological configuration that enables one, by performing an observation and then a measurement to define the state of reality to allows a QT to estimate, probabilistically, the outcomes of future experiments, even without it being possible to track how these outcomes come about.

The arrangement of the double-slit experiment as defined by the diagram with two slits, D2, may be seen in these terms, especially in the interference setup, which, in the present view, is made possible by the entanglement between D2 and the (ontological) quantum object, O, traversing it. In this view, this arrangement is not a device enabling tracing the behavior of quantum objects traversing the diaphragm, but a device enabling the creation of traces, observed in the screen, as a new physical configuration of events, a configuration never encountered in physics before and even previously unimaginable, and yet predictable by QM. An additional novelty introduced in this article is that this creation is enabled by the interaction as the entanglement between an ontological quantum object and an ontologically classical object. It is possible that other experimental configurations of this type may be established (for example, in other paradigmatic quantum experiments) or even designed in future quantum experiments.

Correlatively to this experimental situation, the present interpretation gives new meaning and significance to the concept of event, while distinguishing itself and potentially challenging alternative event-based interpretations, such as relational interpretations or those along QBist lines (there are different versions within each type), in which the concept of decision, central to the present interpretation, does not figure meaningfully. More significantly, none of these interpretations, or any other I am aware of, consider the entanglement between ontologically quantum objects and ontologically classical objects, viewed as experimentally quantum objects. This entanglement supports and reveals new dimensions of the fundamental role, defined by the Bohr postulate, of classical physics in quantum physics, a role advocated by this author on several previous occasions [3,4,5].

The present interpretation of QT and quantum phenomena themselves, including those observed in the double-slit experiment, does not remove the conundrum of the experiment or the mysteriousness of quantum physics, manifested in this conundrum. Indeed, according to the present view of quantum physics, based on this interpretation, removing this mysteriousness *may not* be possible in the sense of ever making it representable or even conceivable how quantum phenomena come about. I am not saying that it *will not be* possible; it might be possible. Given the state of fundamental physics now, it is difficult to predict one way or the other, or even whether quantum physics itself, as we know it now, will survive, for example, in considering gravity, for which there is no quantum theory thus far. Instead, however, seeing this impossibility as a problem, which it is from the realist perspective, RWR interpretations, such as the present one or that of Bohr, see this mysteriousness as solution, in the absence of any mysticism, “incompatible with the true spirit of science” [12] (p. 83) [13] (v. 2, p. 63). These interpretations preserve the mathematical-experimental character of QM, defining its continuity with modern physics, from Descartes and Galileo on. As Bohr said more than half a century ago,

This [RWR] argumentation does of course not imply that, in atomic physics, we have no more to learn as regards experimental evidence and the mathematical tools appropriate to its comprehension. In fact, it seems likely that the introduction of still further abstractions into the formalism will be required to account for the novel features revealed by the exploration of atomic processes of very high energy.

[13] (v. 3, p. 6)

The history of high-energy physics and QFT has confirmed this assessment, made in 1958, and continues to do so, without, thus far, contradicting this argumentation. So has the history of QM during the same period. It is true that, unlike mathematical breakthroughs in QFT, such as those that led to the standard model, there have been no major changes in the mathematics of QM. Even so, the exploration of quantum correlations from the 1960s on and quantum information theory have been major developments, which opened new possibilities for the future of QT, including QFT and possibly beyond.

What is true is that, while preserving the mathematical-experimental character of quantum physics, RWR thinking changes the nature of our thinking and knowledge, thereby making that which is beyond thought part of our thinking and knowledge. As such, however, RWR thinking is not only compatible with but may also be necessary for advancing physics. Thus, although it could not have done so on its own accord, because great physical, mathematical, and philosophical creativity was necessary, this thinking led to Heisenberg’s invention of QM and then the uncertainty relations, to Bohr’s invention of complementarity and his interpretation of QM, and to the thinking of Heisenberg, Dirac, and others in the development of QFT and its “mathematical abstractions.” RWR thinking may continue to do so in dealing with the problems with which fundamental physics confronts us now, even though these problems, too, are likely to require, beyond RWR thinking, the same extraordinary physical, mathematical, and philosophical creativity on which fundamental physics has always depended in moving itself forward. It has never been otherwise. Nor has this article argued otherwise.

## Data Availability

Data is contained within the article.
